# Negative Feedback Governs Gonadotrope Frequency-Decoding of Gonadotropin Releasing Hormone Pulse-Frequency

**DOI:** 10.1371/journal.pone.0007244

**Published:** 2009-09-29

**Authors:** Stefan Lim, Lilach Pnueli, Jing Hui Tan, Zvi Naor, Gunaretnam Rajagopal, Philippa Melamed

**Affiliations:** 1 National University of Singapore, Graduate School for Integrative Sciences and Engineering, Centre for Life Sciences, Singapore, Republic of Singapore; 2 Faculty of Biology, Technion-Israel Institute of Technology, Haifa, Israel; 3 Department of Biological Sciences, National University of Singapore, Singapore, Republic of Singapore; 4 Department of Biochemistry, George S. Wise Faculty of Life Sciences, Tel Aviv University, Ramat Aviv, Israel; 5 The Cancer Institute of New Jersey, New Brunswick, New Jersey, United States of America; Ecole Normale Supérieure de Lyon, France

## Abstract

The synthesis of the gonadotropin subunits is directed by pulsatile gonadotropin-releasing hormone (GnRH) from the hypothalamus, with the frequency of GnRH pulses governing the differential expression of the common α-subunit, luteinizing hormone β-subunit (LHβ) and follicle-stimulating hormone β-subunit (FSHβ). Three mitogen-activated protein kinases, (MAPKs), ERK1/2, JNK and p38, contribute uniquely and combinatorially to the expression of each of these subunit genes. In this study, using both experimental and computational methods, we found that dual specificity phosphatase regulation of the activity of the three MAPKs through negative feedback is required, and forms the basis for decoding the frequency of pulsatile GnRH. A fourth MAPK, ERK5, was shown also to be activated by GnRH. ERK5 was found to stimulate FSHβ promoter activity and to increase FSHβ mRNA levels, as well as enhancing its preference for low GnRH pulse frequencies. The latter is achieved through boosting the ultrasensitive behavior of FSHβ gene expression by increasing the number of MAPK dependencies, and through modulating the feedforward effects of JNK activation on the GnRH receptor (GnRH-R). Our findings contribute to understanding the role of changing GnRH pulse-frequency in controlling transcription of the pituitary gonadotropins, which comprises a crucial aspect in regulating reproduction. Pulsatile stimuli and oscillating signals are integral to many biological processes, and elucidation of the mechanisms through which the pulsatility is decoded explains how the same stimulant can lead to various outcomes in a single cell.

## Introduction

The pituitary gonadotropins, follicle stimulating hormone (FSH) and luteinizing hormone (LH), have distinct roles in regulating gonadal development and function, and thus show different temporal expression, although both hormones are produced in the same cell and their biosynthesis is regulated by the same gonadotropin-releasing hormone (GnRH). The gonadotropin hormones are heterodimers: the α-subunit (αGSU) is common to both hormones, whereas the β-subunit is unique and confers biological specificity. The differential β-subunit gene expression is regulated by differing GnRH pulse-frequency: increasing pulse-frequency stimulates LHβ gene expression, and lowering it results in a decline in LHβ but a rise in FSHβ expression; the expression of αGSU is less stringently regulated and is stimulated by continuous or high frequency GnRH administration [1]–[4]. The mechanisms through which the cell is able to decode the different frequencies of GnRH and translate them into differential subunit gene expression has yet to be elucidated [5].

Previous studies have proposed receptor desensitization as the primary means of differentiating between the frequencies of GnRH pulses, even though the mammalian GnRH-receptor (GnRH-R) is an atypical G-protein-coupled receptor that lacks a carboxyl-terminal domain, and thus exhibits slow internalization and a lack of rapid desensitization [6]–[9]. However, a correlation was reported between GnRH-receptor (GnRH-R) concentration and optimal levels of gonadotropin subunit gene expression under different GnRH pulse frequencies [10], [11]. Receptor concentrations after 20 h GnRH exposure were highest for intermediate GnRH pulses (1 pulse/30 min), coinciding with high levels of αGSU, LHβ and GnRH-R promoter activity, while highest levels of FSHβ promoter activity occurred with lower receptor concentrations at slower GnRH frequencies (1 pulse/2 h; [12], [13]). A direct effect of GnRH on GnRH-R transcription has been shown [11], [14]. It is therefore possible that GnRH regulates differentially the gonadotropin subunit genes through controlling GnRH-R gene expression and cell surface receptor concentration, which would impact downstream signalling events.

On binding the GnRH-R, GnRH triggers a cascade of events resulting in the activation of three major mitogen-activated protein kinase (MAPK) cascades: extracellular-signal regulated kinase (ERK) 1/2, c-Jun NH2-terminal kinase (JNK) and p38. As a result of their phosphorylation, ERK1/2 is activated about 12 fold, JNK 20–50 fold, and p38 about 2 fold [15]. The three gonadotropin subunit genes are activated by different combinations of these MAPK pathways: while all three subunit genes require activated (p) ERK1/2 for transcriptional activation, LHβ also requires pJNK, while FSHβ requires all three MAPK pathways; pJNK also targets GnRH-R gene expression [14], [16]–[18]. A fourth MAPK, Big MAPK (BMK) or ERK5, is also activated by GnRH, but little is known about its effect on gonadotropin subunit gene expression [15]. We have previously shown that GnRH-activated regulation of FSHβ expression involves Nur77 and MEF2, both of which are activated by ERK5 in T-cells [19], [20]. It is therefore conceivable that ERK5 also features in the differential expression of the gonadotropin subunit genes, by regulating specifically FSHβ transcription through Nur77 and MEF2D. Interestingly, Nur77 decreases GnRH-R gene expression [14], [21], raising the possibility that it is also involved in the frequency-decoding by regulating the number of GnRH-Rs.

Concomitant with the GnRH activation of ERK1/2, JNK and p38, their specific MAPK phosphatases (MKPs), dual-specificity phosphatases (DUSP) 1 and 4, are also up-regulated [22]. These dephosphorylate threonine and tyrosine residues on MAPKs, rendering the MAPKs inactive [23]. The pMAPKs enhance both transcriptional activation and protein stabilization of the DUSPs, to provide negative feedback which is fine-tuned by the individual preferences of each DUSP towards a particular MAPK [23].

The dependence of gonadotropin subunit gene expression on the MAPKs has two likely implications on the GnRH frequency-decoding mechanism: firstly, the differential reliance of the three subunit genes on various combinations of the pMAPKs could contribute to GnRH frequency-decoding. The αGSU, which depends only on pERK1/2 for activation, might be optimally expressed at GnRH frequencies at which only ERK1/2, but not JNK or p38 are highly activated. On the other hand, genes requiring more than one or two pMAPKs, would be optimally expressed only at frequencies at which all the requisite MAPKs are activated simultaneously at the highest levels. Such a synchronization of MAPK activation could be dictated by GnRH frequency, and furnish a reasonable connection between GnRH frequency and differential subunit gene expression.

Secondly, MAPK activity thresholding has been suggested as a possible mechanism for differential gene activation [5]. Both the αGSU and FSHβ depend heavily on pERK1/2 for transcriptional activation; this is inactivated by DUSP1 which is also up-regulated by GnRH [22]. Hence, higher frequencies of GnRH might prevent the level of active pERK1/2 from reaching the threshold required to induce FSHβ gene expression, although it may be sufficient for αGSU expression. Given that slower pulses of GnRH would result in less DUSP1 activity, this would allow sufficient build up of pERK1/2 to pass the threshold level [5]. The level of the threshold would depend on the amount of negative feedback by each MKP against its specific MAPK, and the frequency of GnRH pulses would regulate MAPK activity through tuning the extent of negative feedback, thus allowing negative feedback to form the basis of the frequency-decoding.

To date, various experimental approaches used to clarify the molecular mechanisms of frequency-decoding of GnRH by the gonadotrope have provided only partial explanations (e.g. [5], [13], [24]–[26]). Negative feedback was suggested to be involved after several genes encoding various factors known to act as negative regulators of GnRH-activated pathways were seen to be elevated following GnRH exposure [8], [27], [28]. Although key network features that could help in the frequency-decoding, conceptual models and partial experimental evidence have been proffered, the previous studies failed to extend their findings to demonstrate how they explain the differential expression of the three subunit genes.

The aim of this study was to elucidate the frequency-decoding mechanism through use of computational and experimental methods. We initially employed mathematical modelling and computer simulations to test the possibility that MKP-negative feedback, coupled with the differential reliance of the three subunit genes on various combinations of pERK1/2, pJNK and pp38, comprises the basis of frequency-decoding of the GnRH pulses. This also allowed us to demonstrate quantitatively MAPK activity thresholding and define it in mathematical terms. Next, we examined experimentally the role of ERK5 in regulating FSHβ gene expression, and were then able to augment our mathematical model with the experimental findings. Finally, receptor dynamics were incorporated into the model to clarify the role of receptor concentration in regulating differential expression of the subunit genes.

## Results

### MKP negative feedback gives rise to frequency-dependent differential gonadotropin subunit gene expression

**Figure 1:**
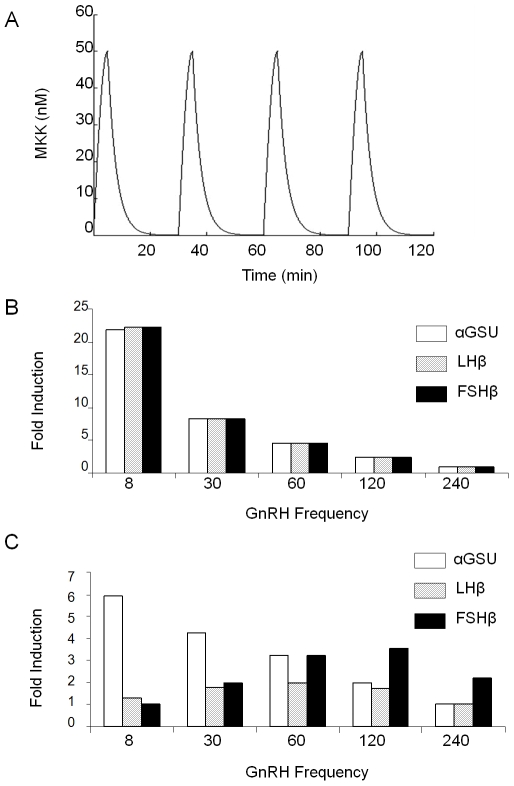
Phosphatase feedback results in differential gene expression. (A) The pulsatile profile of pulses for MAPKK activation used in simulation of models. The pulse increases for 5 min in a sinusoidal fashion to reach its maximum value, before undergoing an exponential decay. (B) The basic model without negative feedback, where DUSP1 and 4 levels were kept constant, was simulated for 1440 min for five different pulse frequencies of the pMAPKK stimulus: 8 min, 30 min, 60 min, 120 min and 240 min. At the end of each simulation, the total accumulated concentrations of the αGSU, LHβ and FSHβ subunits were noted and plotted as fold differences over the lowest concentration for each subunit among the five frequencies tested. (C) The basic model, where DUSP1 and 4 levels were allowed to be actively induced by pERK1/2 and pJNK, was simulated and graphs plotted as in (B).

In order to examine the possibility that negative regulation of MAPKs by their specific phosphatases has a role in the frequency-decoding of GnRH pulses to allow differential gonadotropin subunit gene expression, we constructed a basic model that re-enacted the differential dependence of each subunit on the known combinations of pERK1/2, pJNK and pp38, which included also the MKPs that they activate, and against which these MKPs act. Computer simulations of this basic model were carried out using a pulsatile profile for MAPKK activation (Figure 1A). Each simulation ran for 1440 minutes of simulation time.

The basic model was run at five different frequencies: 8, 30, 60, 120 and 240 min, which reflect the physiologically relevant GnRH pulse frequencies and include those employed in previous studies [2], [13], [29], so allowing comparison with results from the simulations. We first ran simulations of the basic model for each of the above frequencies to obtain the minimum root mean square (rms) values of DUSP1 and 4, as measures of the average activation of these phosphatases. We then replaced the starting concentrations of these phosphatases with their minimum rms values, and re-defined their equations to maintain them at these concentrations throughout the length of the simulation. This prevented any kinase from activating them, and thus any potential negative feedback against an activating kinase. Also, we chose the minimum rms values so that at the lowest frequencies, at which the rate of MAPK activation is slowest, the levels of the MKP would not be so high as to over-damp MAPK activity.

Simulations showed that for all three subunits, highest levels of expression were obtained with 8 min-pulses, and these levels were progressively reduced with decreasing frequency of the stimuli (Figure 1B), indicating a lack of differential gene expression. When the original rate equations governing DUSP1 and 4, together with their previous starting concentrations were restored, highest levels of α-subunit expression were obtained for 8 min pulses, for LHβ at 60 min pulses, and for FSHβ at 120 min pulses (Figure 1C). This demonstrates that the negative feedback by the phosphatases is crucial for the differential expression of the gonadotropin subunit genes.

To determine if the model is robust and whether the positive results obtained are unique to a single set of parameter values, a sensitivity analysis was carried out. Each kinetic parameter was adjusted in turn by 10% of its original value, and the trends of gonadotropin subunit expression with various frequencies were noted as before. The distinct differential gene expression was maintained throughout the changes in each of the kinetic constants perturbed for the basic model with feedback. Similarly, the lack of differential gene expression was observed for all variations of each kinetic constant for the model without feedback (Supplementary Figure S1).

### Differential gene expression results from a phosphatase-induced increase in average MAPK activation with decreasing frequency of the stimulus

Since negative feedback on the MAPKs appears critical for differential gene expression, we examined the nature of the effects of these phosphatases on the pMAPKs. For this, we looked at the maximum amplitude, the rms value and the total amount of activated kinase for each pMAPK. Fold differences for each of these quantities for each pMAPK were then plotted.

**Figure 2:**
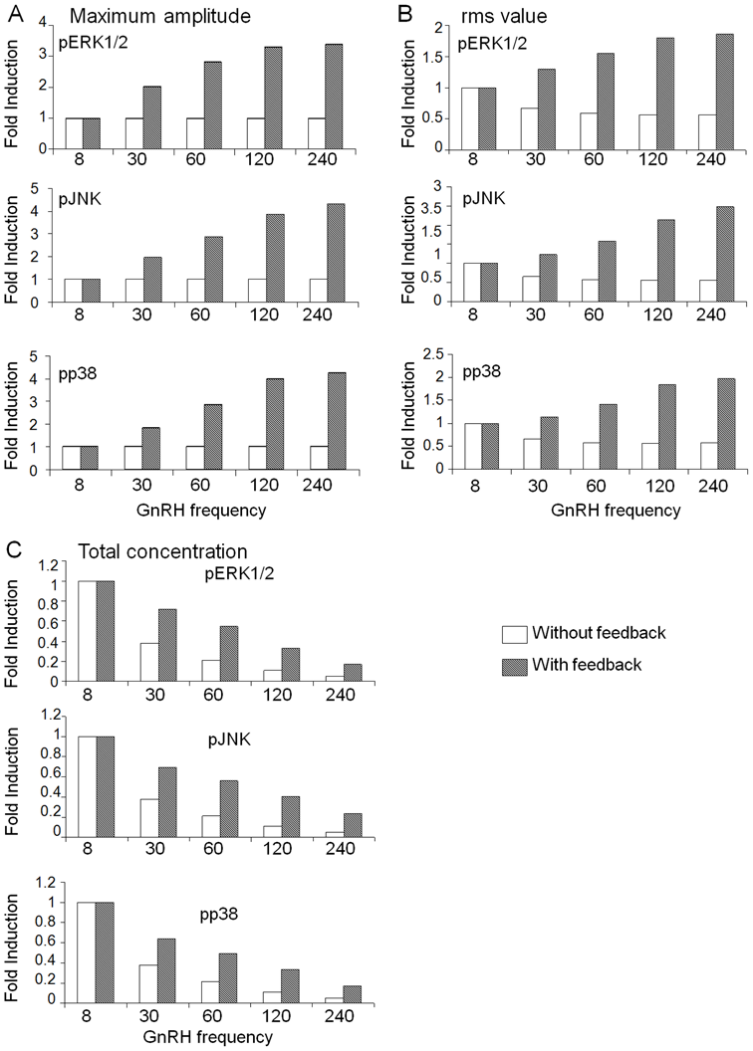
Analysis of MAPK activation for the basic model. The basic model with and without phosphatase feedback was simulated as in Figure 1. At the end of each simulation, (A) the maximum steady-state amplitudes, (B) the root mean square (rms) values, and (C) the total concentrations of each activated (p) MAPK were computed. These values were plotted as fold differences over corresponding values for 8 min pulse-frequency.

In the absence of negative feedback, the maximum amplitude was the same for all frequencies for each pMAPK (Figure 2A). On the other hand, the rms values declined gradually with decreasing frequency (Figure 2B). The total amount of activated kinase over the 1440 min of simulation time dropped starkly with decreasing frequency (Figure 2C). Although in the presence of negative feedback, there was also a steady decrease in the total amount of activated kinase, this was not as sharp as when negative feedback was lacking (Figure 2C). Moreover, both the maximum amplitude and the rms value for each activated kinase increased steadily with decreasing frequency (Figures 2A and B). These results suggest that differential gene expression requires an increase in rms value with decreasing GnRH frequency caused by the negative feedback from the phosphatases.

### GnRH activates ERK5 which stimulates FSHβ and down-regulates GnRH-R gene expression

**Figure 3:**
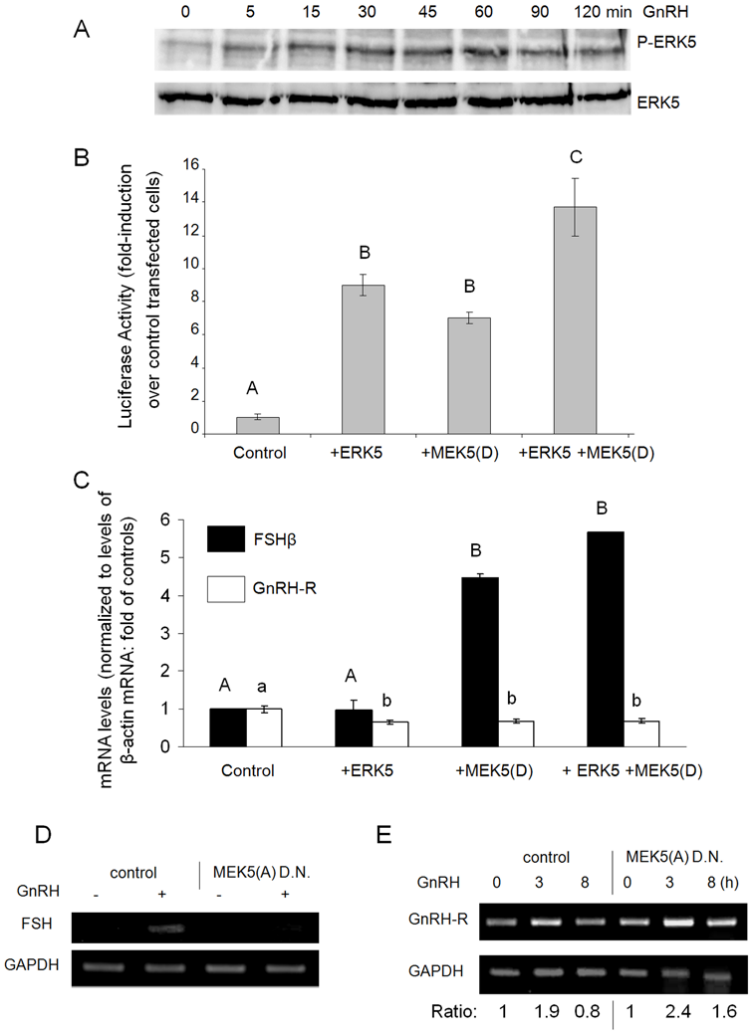
GnRH increases FSHβ gene expression through activation of ERK5. (A) LβT2 cells were cultured and exposed to GnRH for 0–2 h, before lysis and western analysis using pERK5 (upper panel) or total ERK5 (lower panel) antisera. (B) The mouse FSHβ-luc construct (200 ng), and ERK5 or constitutively-active MEK5(D) expression vectors, or both (50 ng each) were transfected into αT3-1 cells in 96-well plates. Luciferase levels were normalized to those of Renilla, and results show the fold induction over untreated FSHβ-luc control-transfected cells. Mean±SEM, n = 6. ANOVA followed by Bonferroni t-test compared means; those not significantly different (p>0.05) are designated the same letter. (C) Cells were cultured in 6-well plates and transfected with 2 µg of ERK5 or MEK5(D) expression vectors or both; after 48 h, RNA was extracted for RT-PCR. Primers amplified 856 bp of FSHβ, 200 bp of GnRH-R, or 200 bp of β-actin cDNA as control. The amplicons were run on an agarose gel, quantified by densitometry analysis, values normalized over those of β-actin and fold differences over control cells plotted. Mean±SEM, n = 3. Statistical analysis (as in Figure 3B) was carried out separately for FSHβ (upper case) or GnRH-R (lower case). (D) LβT2 cells in 60 mm plates were transfected with 4 µg of the dominant negative MEK5(A) construct 24 h prior to GnRH treatment for 8 h. RNA was extracted for RT-PCR; primers amplified the first 225 bp of FSHβ or 230 bp of the GAPDH cDNA, as control. (E) Similarly, cells were transfected with the MEK5(A) construct before GnRH treatment for 3 or 8 h, after which the RNA was extracted, reverse-transcribed and primers amplified a fragment from the GnRH-R cDNA, or GAPDH, as control. The ratio of the GnRH-R amplicon, after normalization with GAPDH, relative to levels in untreated samples (with or without MEK5(A) is noted.

Based on reports that ERK5 activates Nur77 in T-cells [19], and our findings that Nur77 plays a crucial role in FSHβ gene expression [20], we investigated the role of ERK5 in activating FSHβ transcription. We first carried out a time-course analysis of ERK5 activation by GnRH, through western analysis of whole cell lysates from gonadotrope LβT2 cells treated with 100 nM GnRH for 0–120 min. The level of phosphorylated ERK5 (pERK5) clearly increased within 5 min of GnRH treatment, peaking after 30–60 min, and elevated levels were still detected after 90–120 min (Figure 3A).

Having established that GnRH activates ERK5, we carried out promoter activity assays to determine whether the ERK5 is able to increase FSHβ promoter activity. Expression vectors for ERK5 and its activating kinase, MEK5(D), were transfected either individually or together, and the effects on the murine FSHβ promoter-luciferase reporter construct were measured. Transfection of ERK5 or MEK5(D) expression vectors alone induced FSHβ promoter activity 7–9 fold, indicating some basal activity of MEK5 in these cells, possibly due to factors in the serum. However over-expression of both factors together induced activity nearly 14-fold over the levels in untreated cells (Figure 3B).

The ability of pERK5 to affect FSHβ transcription was confirmed using semi-quantitative RT-PCR. Changes in GnRH-R mRNA levels were also measured to assess the possibility that a change in GnRH-R expression also comprises a mechanism for GnRH- and ERK5-induced FSHβ gene expression. The ERK5 alone had no effect on FSHβ mRNA levels, indicating a lack of basal activation of the pathway under these conditions, but it further enhanced the effect of MEK5(D). All treatments marginally decreased GnRH-R mRNA levels, suggesting that GnRH might act through this pathway to down-regulate its own receptor (Figure 3C).

To verify whether the GnRH effect on FSHβ gene transcription is indeed via activation of ERK5, 24 h before GnRH treatment, a MEK5(A) construct that encodes a dominant negative MEK5, was transfected in order to prevent activation of ERK5. RT-PCR analysis showed that the stimulatory effect of GnRH on the FSHβ transcript levels was virtually abolished following this repression of ERK5 activation (Figure 3D). Similarly, the role of ERK5 in GnRH down-regulation of the GnRH-R was tested by transfecting the MEK5(A) construct followed by 3 or 8 h GnRH exposure. After 3 h GnRH exposure, GnRH-R mRNA levels were elevated, but these had returned to basal levels by 8 h exposure. However in the MEK5(A)-transfected cells, the drop at 8 h was clearly reduced (Figure 3E).

### pERK5 enhances FSHβ expression levels in a concentration-dependent manner

**Figure 4:**
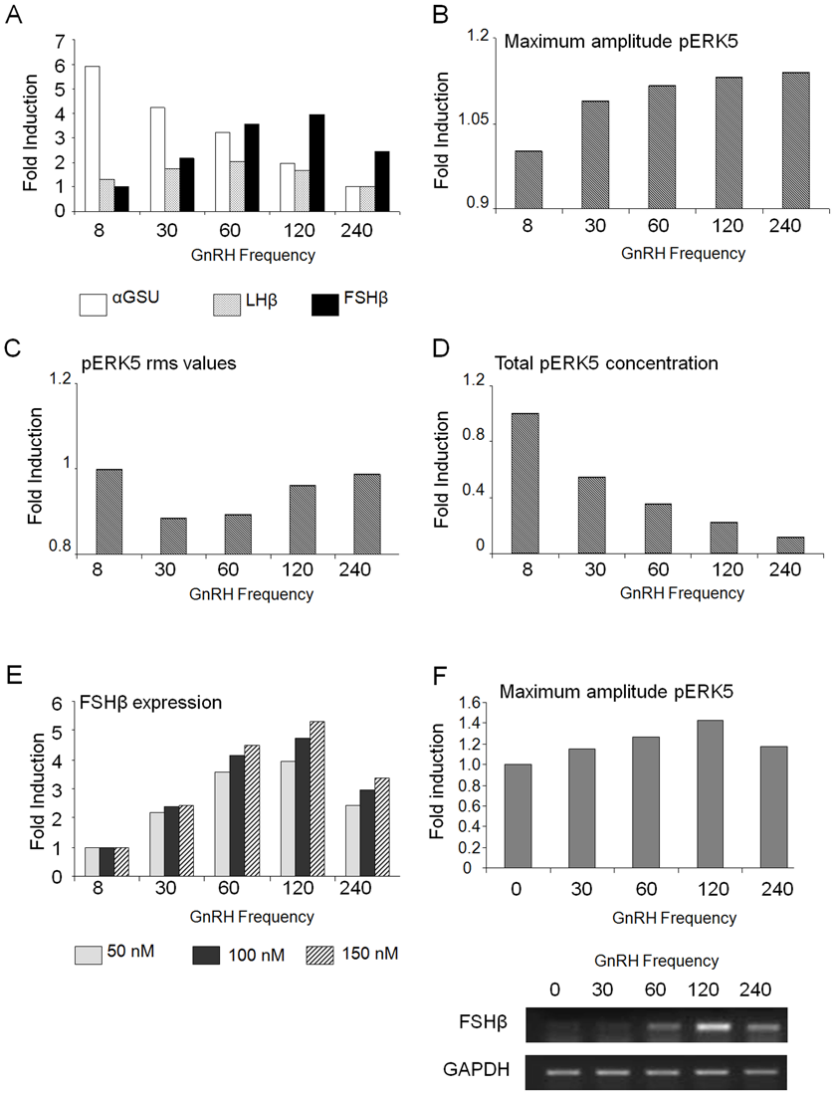
Expanded model with phosphatase feedback demonstrates differential gene expression, which is enhanced by ERK5. (A) The default expanded model with phosphatase feedback was simulated for 1440 min for the same five frequencies and expression trends for each subunit were plotted as described in Figure 1. Thereafter, (B) the maximum steady-state amplitude, (C) the rms value, and (D) the total concentration of activated ERK5 were computed and plotted. (E) The expanded model was then re-simulated with various concentrations of total ERK5 (50, 100 or 150 nM), and the expression trends of FSHβ were plotted. (F) To validate these models, maximum amplitude pERK5 was measured in cells after administering 5 min GnRH pulses at the marked frequencies for 4 h. Protein was collected at 0–90 min after the last pulse and analyzed, together with an internal standard for comparisons, by western blotting for pERK5 and total ERK5. The maximum amplitude for each pulse-frequency is shown after normalization to total ERK levels and to the internal standard. Also shown in the bottom panel are the FSHβ mRNA levels after the last pulse.

Having shown that pERK5 increases FSHβ gene expression, we added this effect to our basic model with phosphatase feedback to form an expanded model. Simulation of this model again revealed differential subunit gene expression. The αGSU was preferentially expressed at 8 min pulse-frequency, LHβ at 60 min, and FSHβ at 120 min (Figure 4A), which was confirmed in the sensitivity analysis (Supplementary Figure S2). Notably, the highest fold induction of FSHβ was greater for the expanded model as compared to the basic model (Figure 4A and Figure 1C), indicating that the pERK5 component in this model boosts levels of FSHβ with decreasing GnRH pulse-frequency. The relative concentrations of the various kinases used in the model, meant that the concentration of ERK5 was the limiting factor, which limited the degree of increase in FSHβ mRNA levels.

The maximum amplitude of activated ERK5 increased with decreasing GnRH frequency, as previously (Figure 4B). The rms value for pERK5 was highest at 8 min pulse-frequency, but dropped slightly with 30 min pulses (Figure 4C). However, it then increased with decreasing frequencies to achieve a level near that of the 8 min pulse-frequency. As with the other MAPKs, the total amount of activated ERK5 over the course of 1440 min of simulation time decreased with lower GnRH frequencies (Figure 4D).

We tested this expanded model with various concentrations of total ERK5 (50, 100 and 150 nM), with 50 nM as the basis for comparison. Simulation results revealed that the induction of FSHβ increased with increasing concentrations of ERK5 (Figure 4E), confirming that the concentration of ERK5 was the limiting factor in the FSHβ response. Hence, ERK5 has the distinct effect of enhancing FSHβ expression, while maintaining its preferred low stimuli-pulse-frequency for optimal expression.

Finally we validated the effect of GnRH pulse-frequency on ERK5, by examining the maximum amplitude of pERK5 after administering GnRH at various pulse frequencies, and measuring protein levels over the next 90 min. The maximum level of pERK5, calculated relative to total ERK5 and normalized with levels of a reference sample, was significantly higher in cells receiving pulses at 120 min intervals than in those receiving pulses at 30 min intervals (1.52±0.06 fold, n = 3; p<0.05). This coincided with the induction of FSHβ mRNA levels which was maximal after 120 min interval pulses (Figure 4F).

### Differential GnRH-R concentration alone appears not to give rise to full differential gonadotropin subunit gene expression

Given that cell-surface GnRH-R concentration was previously reported to correlate with the differential expression of the gonadotropin subunit genes [13], we examined whether differences in GnRH-R at various GnRH pulse frequencies would be sufficient to give rise to differential subunit gene expression. For this, the model was further expanded to include GnRH-R dynamics. Since JNK is reported to up-regulate GnRH-R levels [14] and ERK5 likely down-regulates GnRH-R expression through Nur77 (Figures 3C, 3E and [21]), two tuneable parameters, ε and γ, were introduced to allow us to observe the influence of both pJNK and pERK5 on frequency-decoding through regulating the levels of the GnRH-R.

The model was simulated, firstly without receptor synthesis or degradation, in order to assess the effect of varying receptor concentrations on subunit gene expression. For each pulse-frequency, changes were made in the initial concentration of the free receptor, R, to a factor multiplied by the basal value of 0.01 nM, in accordance with the reported fold stimulation of GnRH-R promoter activity [13]. Hence, for 8 min pulses, this initial concentration would be 0.016 nM, for 30 min pulses 0.018 nM, for 60 min pulses 0.019 nM, for 120 min pulses 0.015 nM, and for 240 min pulses 0.01 nM.

**Figure 5:**
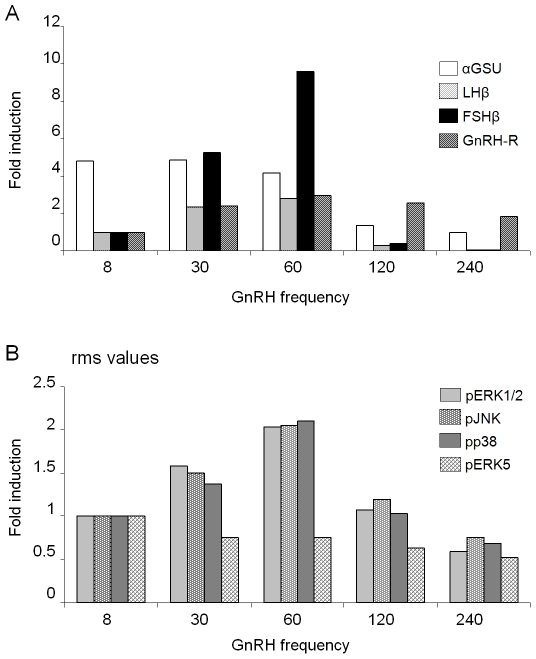
Differential GnRH-R concentration alone does not give rise to full differential gonadotropin subunit gene expression. The receptor-enhanced model was simulated for 1440 min with the total receptor concentration kept constant. For each pulse-frequency, the initial concentration of the free receptor, R, was changed to a factor multiplied by the basal value of 0.01 nM, in accordance with the fold stimulation of GnRH-R promoter activity as reported in the literature [13]. (A) Fold differences for the expression of each subunit, as well as for GnRH-R, were then calculated and plotted as in Figure 1B. (B) Fold differences of the rms value of each activated MAPK were computed and plotted.

Simulation results showed total GnRH-R concentration amassed over the 1440 min, in concurrence with published data [13]. However, while the αGSU was expressed at almost equally high levels for 8–30 min pulses, both the β-subunits had peak expression at 60 min pulse-frequency (Figure 5A). Upon examining the rms values of the pMAPKs, these increased steadily with decreasing frequency until 60 min, after which they decreased (Figure 5B), due to the initial conditions imposed, in which lower concentrations of GnRH-R were present when the model was simulated at 120 min and 240 min GnRH pulse frequencies. Finally, total MAPK activation decreased with decreasing frequency, as before (data not shown).

### JNK-positive feedforward without ERK5-negative feedback on GnRH-R expression causes loss of differential gonadotropin subunit gene expression

**Figure 6:**
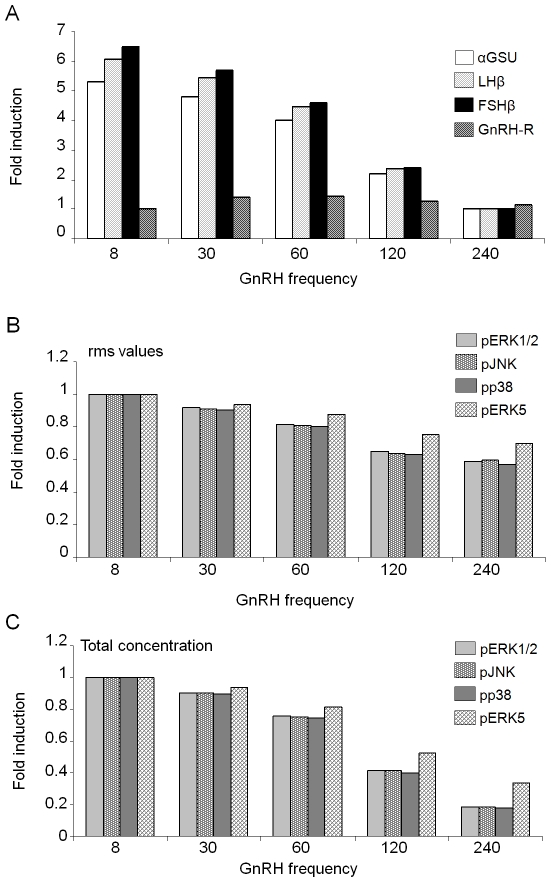
JNK-positive feedforward without ERK5-negative feedback on GnRH-R expression results in loss of differential gene expression. The receptor-enhanced model was simulated for 1440 min. The model was set with ε = 1 and γ = 0, so that the system was deprived of the ERK5-negative regulation of the GnRH-R expression levels. Thereafter, (A) expression trends of each subunit and GnRH-R, (B) rms values and (C) total concentration of each pMAPK were plotted.

The role of the JNK-positive feedforward on GnRH-R expression was investigated in the full model by introducing receptor synthesis and degradation, rather than artificially setting the initial concentration of R for each GnRH frequency. Setting ε = 1 and γ = 0, so that only the JNK-feedforward was permitted, resulted in a total loss of differential gene expression with exponential pulses (Figure 6A). This is despite the model giving the right expression trends for GnRH-R (Figure 6A, compare with Figure 5A).

To understand the possible reasons behind this loss of differential gene expression, both the rms values and the total activation of all the pMAPKs were examined. Unlike before, both these values decreased in tandem with decreasing frequency of the pulsatile stimulus (Figures 6B, 6C). It appears then that while JNK-feedforward may increase GnRH-R levels at lower frequencies, it indirectly also increases the levels of DUSP1 and 4 through greater MAPK activation as a result of the increased receptor concentration, thus the average levels (as given by the rms values) of MAPK activation decrease accordingly. Hence, while there is a correlation between receptor concentration and differential expression of the subunit genes, it is clearly not a straightforward causal relationship.

### ERK5-negative feedback against GnRH-R expression restores differential gonadotropin subunit gene expression in the full model

**Figure 7:**
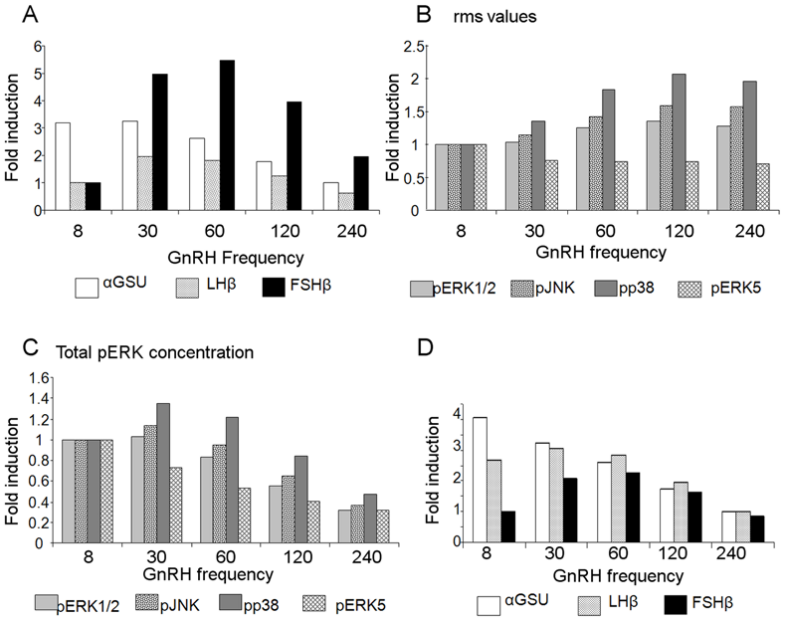
ERK5-negative feedback on GnRH-R expression restores differential gonadotropin subunit gene expression. The receptor-enhanced model was simulated for 1440 min for the same five GnRH frequencies as before. The model was set with ε = 0 and γ = 1, so that the system possesses ERK5-negative regulation, but not JNK-positive regulation of GnRH-R expression levels. Thereafter, (A) expression trends of each subunit, (B) rms values and (C) total concentration of each pMAPK were plotted. (D) Thereafter, the model was set with ε = 1 and γ = 1, so that the system possesses both ERK5-negative regulation and JNK-positive regulation of GnRH-R expression levels.

Having established that the JNK-feedforward on the GnRH-R abolishes differential subunit gene expression, the parameter settings were modified to ε = 0 and γ = 1 in order to investigate the effect of the ERK5-negative feedback against the GnRH-R. This negative feedback was suggested by the earlier finding that ERK5 over-expression reduces levels of GnRH-R mRNA (Figures 3C, 3E), as well as reports that that Nur77, which is activated by GnRH in immature gonadotropes and ERK5 in T-cells, down-regulates GnRH-R expression [19]–[21]. Simulation of the model with these parameter settings restored differential gene expression (Figure 7A). Additionally, even though the increase of pERK1/2, pJNK and pp38 rms values with decreasing frequency was less steep, the decrease in total MAPK activation also decreased less sharply across the frequencies (Figures 7B, 7C), so that FSHβ attained a peak 5.5-fold induction, higher than in the basic or the intermediate models. This suggests that with the feedback against the GnRH-R by ERK5, there is enhancement of the differential effect on FSHβ gene expression. Thereafter, both JNK-feedforward and ERK5-negative feedback were combined by setting both ε and γ = 1, and while peak fold-induction of FSHβ dropped, there was clearly differential gene expression (Figure 7D). As before, sensitivity analyses were carried out to ascertain that the full model, resulting from the inclusion of new kinetic constants and molecular species in the basic and intermediate models, was robust (Supplementary Figures S3, S4, S5).

## Discussion

**Figure 8:**
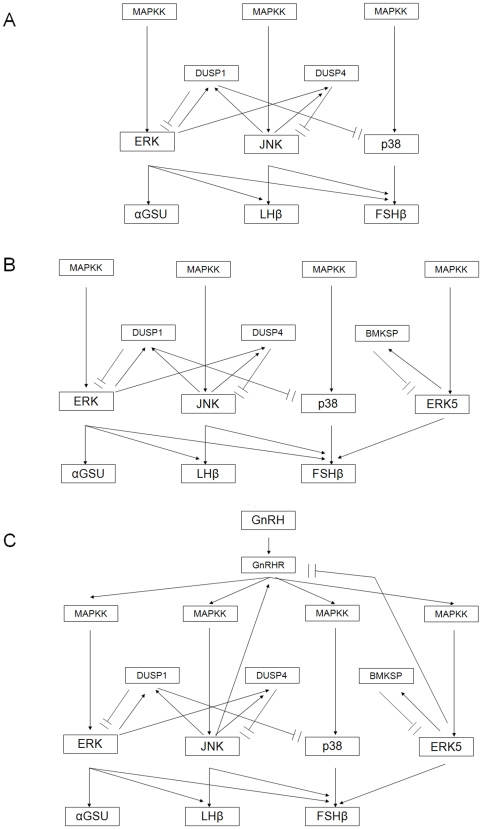
Schematic representation of the models used. The (A) basic, (B) intermediate and (C) full models used in examining the effect of different feedbacks on the decoding of GnRH pulse frequencies for differential gonadotropin subunit-gene expression are shown. Arrows indicate activation (in the case of enzymatic reactions) or induction (in the case of genes). Analogously, barheads indicate de-activation (enzymatic reactions) or repression (gene expression).

The ability of the pituitary gonadotrope to decode GnRH pulse-frequency and differentially regulate gonadotropin gene expression is a crucial regulatory mechanism in reproductive physiology and, given the abundance of hormones secreted in a pulsatile-manner, likely represents a common mechanism in regulatory biology. In this study, we have built a mathematical model that describes the main architecture of the three major GnRH-activated MAPK pathways and have used and refined it, based on original and published experimental evidence, to suggest a mechanism for frequency-decoding. Pivotal to the differential gene activation in this model is the negative feedback on the MAPKs by their specific MKPs (Figure 8).

The negative feedback directed by the MKP makes the maximum amplitudes and rms values of each pMAPK sensitive to changes in GnRH pulse-frequency. The reason for the former can be explained from the model equations, where the dynamics of each MAPK are governed by two factors: induction by pMAPKK and dephosphorylation of its active form by a MKP. The concentration of pMAPKK, with our model parameters and initial concentrations, always reaches a peak of 50 nM with each pulse, so that for each cycle, it activates MAPK to a similar maximum regardless of frequency. On the other hand, MKP activation depends on its activating pMAPKs, whose concentrations fluctuate with frequency and time, where higher frequencies of the stimulus mean greater amounts of pMAPK. Hence, the maximal activation of a MAPK is frequency-dependent and this is supported experimentally [25]. Similarly, the rms value of each pMAPK is also frequency-sensitive through its dependence on both the maximum amplitude of MAPK activation attained, and the frequency of the stimulus, since it involves computing directly the area under the curve depicting total MAPK activation. This leads us to support the theory of frequency-decoding based on MAPK activity thresholding [5].

To define these thresholds, the rate equations governing the synthesis of each gonadotropin subunit mRNA can be generalized as:
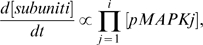
where i = 1 for the α-subunit, i = 2 for LHβ and i = 3 for FSHβ ( = 4 for the expanded models). Such a use of transcriptional logic has already been successfully carried out for prokaryotic systems, and is likely valid for eukaryotic systems [30]. Using multivariate differential calculus, we can determine a critical set of concentrations of the component pMAPKs, which gives rise to the maximal rate of mRNA synthesis. Continuity of these rate functions then implies the existence of a threshold set of concentrations lower than this critical set, above which the synthesis rate remains over a pre-determined level. Frequency-decoding arises from singling out frequencies that are able to cause activation of the component MAPKs consistently above this threshold. Frequencies that bring about highest rms (average) activation and peak activation of component MAPKs are optimal for subunit gene expression. This is apparent for FSHβ at low GnRH frequencies, where negative feedback contributes to the high rms and peak activation values at these frequencies. On the other hand, high GnRH frequencies favor αGSU mRNA synthesis, presumably because the total amount of ERK1/2 activation achieved with these frequencies outweighs the less significant reduction in both rms and peak activation. It appears, therefore, that the greater the number of MAPK dependencies, the more important the roles of rms and peak activation, and hence the negative feedback.

Thresholding and its role in determining gene regulation have been studied extensively in developmental biology, particularly in the context of pattern formation. During development, cells that are fundamentally identical and differ only by their location in relation to the stimulant, respond differently to varying concentration of the stimulant, thus enabling concentration- and position-dependent responses to morphogens for appropriate re-programming of transcription to effect correct speciation [31]–[34]. Also in enzymatic cascades, thresholds, together with negative feedback, have been described as giving rise to time lags leading to mitotic oscillations. Moreover, a mechanism for the origin of the thresholds was proposed in terms of the phenomenon of zero-order ultrasensitivity as described for biochemical systems regulated by covalent modification [35]. Through our proposed correlation between the rates of gonadotropin subunit mRNA synthesis with concentrations of pMAPKs, ultrasensitive behavior becomes embedded within the product of pMAPK concentrations and the higher the number of pMAPK dependencies, the greater the ultrasensitivity.

The mechanism of frequency-decoding in the gonadotrope was further clarified by the novel elucidation of the role of ERK5 in FSHβ expression. Notably the activation kinetics of ERK5 by GnRH are similar to those of ERK1/2 [17], while its low levels give an indication of the typical amounts of pERK5 in these cells. These findings justify the incorporation of ERK5 into our model, in order to test its role in GnRH-frequency-decoding. The kinetic constants and initial concentrations applied to pERK5 in this enhanced model are in agreement with our experimental findings. Since we possess little information on the regulation of ERK5 activity, in particular to its specific-phosphatase and the regulation of this phosphatase, we have hypothetically defined an ERK5-specific phosphatase (BMKSP), which is regulated solely by pERK5, and feeds back to negatively regulate pERK5. Based on how other MAPKs are regulated, we believe it is highly probable that such a phosphatase exists [23]. Thus, ERK5 differs from the other MAPKs in our models, in that it is autonomously regulated, through its unique phosphatase. The ERK5-specific phosphatase, and hence ERK5 itself, are likely however, to be regulated by other kinases, but not ERK1/2, JNK or p38 [23], [36]. Therefore, the autonomy of ERK5 regulation, in the context of our models, is valid.

Simulation of this expanded model confirmed that with the inclusion of ERK5 and its phosphatase, differential gene expression is maintained. With the feedback mechanism on all four pMAPKs in place, the maximum amplitude and rms value of pERK5 behave similarly to those of the other pMAPKs (cf. Figures 2 and 4), demonstrating appropriate feedback behaviour. The inclusion of ERK5 and its inductive effect on FSHβ mean that the rate of FSHβ mRNA synthesis is proportional to the product of the concentrations of the four pMAPKs. This results in a slight increase in the amounts of mRNA synthesized as compared with the basic model (4 fold vs 3.5 fold) at 50 nM ERK5. The increase is small because of the difference in one order of magnitude between the total concentration of ERK5 and the rest of the MAPKs, thus making it the rate-determinant. Ultrasensitive behaviour was observed as we ran the expanded model with other values of total concentration of ERK5 that were closer to those of the other MAPKs. The fold difference increased from 4-fold to close to 6-fold when the ERK5 was increased from 50 nM to 150 nM. Hence, ERK5 increases the ultrasensitive behaviour of FSHβ expression, and in so doing, both increases its level of expression, as well as stabilizing its preference for low frequencies of the stimulus. Moreover, the maximum amplitude of pERK5 predicted by the model at the slower pulse frequencies was validated experimentally and co-incided with the greatest increase in FSHβ mRNA levels (Figure 4F).

GnRH regulation of GnRH-R transcription, which is also dependent on GnRH pulse-frequency, is at least partially through JNK-mediated stimulation, and through Nur77-mediated repression [10], [11], [14], [21]. This indicates a possible role for JNK and ERK5, which activates Nur77 in other contexts [19] and was seen to reduce GnRH-R mRNA levels (Figure 3D), also in the frequency-decoding of GnRH signals. Having demonstrated a role for pERK5 in GnRH down-regulation of GnRH-R (Figure 3E), we added this effect of GnRH to the model. Initially, by keeping the total concentration of GnRH-R for each of the pulse frequencies at the reported levels of GnRH-R promoter activity [13], the correct expression profile for GnRH-R was seen, but there was reduced differential expression of the subunit genes (Figure 5).

Simulation with JNK-induction, but without ERK5-inhibition of GnRH-R demonstrated an appropriate expression profile for GnRH-R, but a loss of differential gene expression. There was also a decrease in both the rms values and total activation of all pMAPKs with decreasing pulse-frequency. We consider it likely that JNK induces GnRH-R at lower GnRH pulse frequencies, but that the consequent increase in MAPK activity from elevated receptor numbers also increases phosphatase activity, which significantly lowers the MAPK activity even below the basal levels. However, the introduction of pERK5 to this model restored differential gene expression, even though ERK5 activation down-regulates GnRH-R expression levels (Figure 3C, E). This suggests that pERK5 helps to modulate the JNK-induced decline in MAPK activity by controlling the levels of GnRH-R, so that the MAPKs can be activated in a fashion that allows frequency-decoding for differential gene expression. This likely comprises an additional role of ERK5 in the process of frequency-decoding. It appears then, that while the levels of GnRH-R may be correlated with the optimal expression of each of the subunits [13], simply increasing or decreasing receptor numbers may not actually bring about complete differential gene expression. It is important that these fluctuations in receptor numbers are controlled by specific agents (pERK5 and pJNK in this case) in a specific way (JNK positive feedforward, ERK5 negative feedback), so that the receptors can, in turn, activate the MAPKs appropriately to enable differential gene expression and frequency-decoding.

Pulsatile stimuli and oscillating signalling messengers are a common feature governing many biological processes (e.g. [37]–[39]). Elucidation of the mechanisms through which the pulsatility of signals is decoded by the cells explains how the same stimulant can lead to various outcomes in a single cell. In this study we have taken a modular approach in order to produce a model of the signalling in the gonadotrope cell, which is computationally accurate due to its foundation in experimental data. This approach is likely to be more accurate than trying to incorporate information regarding the entire network, much of which is irrelevant and likely inaccurate due to a large amount of kinetic parameters that need to be estimated [40]–[42]. Our model predicts a crucial role for MKP feedback and incorporates also a novel role for ERK5 which we have shown experimentally to be relevant, while the changing number of GnRH-Rs on the cell surface appears to be less significant in the frequency-decoding. While this is an important finding in understanding regulation of the pituitary gonadotrope in the context of reproductive physiology, resolution of the mechanisms involved in frequency-decoding contribute to a deeper conceptual understanding of the mechanisms governing differential gene expression in regulatory biology.

## Materials and Methods

### Cell culture and transfections

Experiments were carried out in αT3-1 and LβT2 murine gonadotropes which were cultured and transfected at 50–60% confluence using GenePORTER 2 (Gene Therapy Systems, San Diego, CA) transfection reagent, as described previously [43]. For RT-PCR analysis, the LβT2 cells were cultured in dialysed FCS (Biological Industries, Bet HaEmek, Israel), which optimized the GnRH response. As appropriate, cells were exposed to 100 nM GnRH (Busserelin; Sigma; dissolved in H_2_O) which was added at a volume of 0.1% of the culture medium. The ERK5, MEK5(A) and MEK5(D) expression constructs (gifts from Astar Winoto, UC Berkeley) were transfected at 2 µg per well in six-well plates or 4 µg per 60 mm plate, and total amounts of transfected DNA were equilibrated with pWS.

Reporter gene assays were carried out using 600 bp of the proximal murine FSHβ gene promoter fused to the firefly luciferase gene, as described previously [44]. Firefly luciferase values were normalized to those of Renilla luciferase which was co-transfected as an internal control. Experiments were carried out on at least three separate occasions, and representative results are shown.

### RNA extraction and reverse transcriptase PCR

RNA was extracted using TRIzol reagent (Invitrogen, Carlsbad, CA), and the total RNA (5 µg) was reverse transcribed using Moloney murine leukemia virus (Promega, Madison, WI) reverse transcriptase and oligo(dT) primers (5 mM; New England Biolabs, Beverly, MA). PCR amplification was carried out using primers, as indicated in the figure legends. Amplification of mouse β-actin or GAPDH served as an internal control. All samples were assayed in duplicate.

### Western blot analysis

Western blot analysis was carried out as previously described [44] using antisera targeting phosphorylated ERK5 (pERK5) and total ERK (Cell Signaling Technology).

### Computational modeling of the GnRH network

To model the general topology of the GnRH-R-stimulated signaling network, we assume that each activating kinase, pMAPKK, has an activation profile mimicking that of the pulsatile GnRH stimulus, differing only in amplitude. We also assume that the phosphatases involved act directly at the level of the MAPK and not the MAPKK [23], [36]. Each activating MAPKK acts on an unphosphorylated MAPK to yield the phosphorylated (p) MAPK, which is subsequently dephosphorylated by the relevant MKP. Applying first-order Michaelis-Menten kinetics with turnover numbers kcat_1_ and kcat_−1_, and Michaelis constants, km_1_ and km_−1_, we can represent this by:
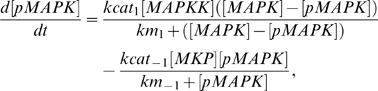
where ([MAPK] - [pMAPK]) denotes the amount of unphosphorylated MAPK remaining at any one time. The values of the kcat_1_, km_1_, kcat_−1_ and km_−1_ have all been adapted from the Database of Quantitative Cellular Signaling (DOQCS) [45], as the basic kinetic constants for the phosphorylation and de-phosphorylation of ERK, and are provided in Supplementary File S1 and in Supplementary Tables S1, S2, S3, S4.

The phosphatases are up-regulated by their respective kinases as documented in the literature, and this is expressed as simple proportions of these kinases. The basic rate of DUSP1 activation has been taken from DOQCS. Moreover, as the induction of DUSP4 is much slower as compared to DUSP1 [22], the rate of DUSP4 induction by ERK1/2 is reduced to 20% that of DUSP1. Their degradation is proportional to their instantaneous amounts. This gives:
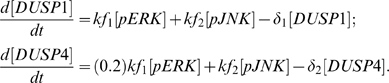



The rate of change of the amounts of each gonadotropin subunit mRNA is made proportional to the product of the amounts of their requisite pMAPKs. This will allow us to test whether GnRH frequencies indeed synchronize the periods of highest activity for the various MAPKs for optimal subunit expression. If this is not the case, and these MAPKs are asynchronously-activated, then the product of their amounts would remain relatively stable with time, without peaking significantly. The consequence of this would be the lack of unique frequency regimes where each gonadotropin subunit is optimally expressed. We thus have:
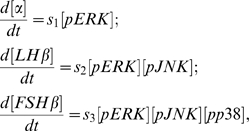
where 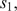 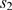 and 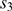 are arbitrarily chosen, without any ill-effect on the overall behavior of each gonadotropin subunit gene. The above equations thus form the basic model.

To expand the basic model, we add equations governing the phosphorylation and de-phosphorylation of ERK5 by its specific phosphatase, and modify the expression for FSHβ to include ERK5:
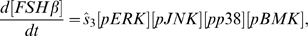
where 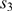 has been re-scaled to 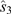 to fit in the fourth variable.

The activation profile of the MAPKK used as a stimulus for the model is a pulse that peaks after 5 min in a sinusoidal fashion, followed by an exponential decay with rate κ for the inter-pulse duration dictated by pulse-frequency of GnRH.

For inclusion of receptor dynamics, a published model was utilized up to the formulations for intra-cellular calcium (CAC) [46]. The equation governing the free GnRH-R was modified to include expressions for JNK induction and ERK5 down-regulation. To bridge this addendum to the basic model, we assume that MKK (MAPKK) follows the same activation profile as CAC. This is reasonable, given that CAC activates PKC, which is the upstream activator of the various MAPK cascades in gonadotrope cells [15]. Nevertheless, because [CAC] ranges between 0.1 and 1 µM, we multiply it by a factor of 50 and re-assign its unit as nM to convert [CAC] to [MKK] of the basic model. Alternatively, we can co-multiply [CAC] by 50 nM and 1 µM^−1^ to effect the same conversion, but without the need for a re-assignment of units.

The ordinary and delayed differential equations of the mathematical model were converted to a Matlab code and run on Pentium M notebook computer, using Matlab 7.0.4 with either the ode23 or ode23s solver. A number of key readouts at the end of each simulation run were made. Firstly, as a measure of gonadotropin subunit gene expression, the concentration of each subunit at the final time-point was taken. Since no degradation has been introduced for them, this quantity represents the accumulated amount of subunit mRNA produced. Secondly, for the basic and expanded models, the maximum steady state amplitude of each pMAPK was noted. This allows us to observe the impact of the various phosphatases on the activation of each MAPK. However, this was not possible for the full model because the total amount of GnRH-R is always changing, so that the levels of the activated MAPK never reach a steady state. Thirdly, we calculated the root mean square (rms) value of each activated MAPK. Since the activated MAPKs all fluctuate with the frequency of the stimulus, the rms value provides a good estimate of the average activation of each MAPK. Additionally, calculating the rms value for both the free and ligand-bound receptors gives a reasonable approximation of the average concentration of receptors. The rms value for any quantity is given by:
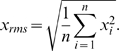



Finally, the total amount of MAPK activated throughout the duration of simulation is given by the area under the solution curve for each of the pMAPKs. Since there is no explicit analytical solution for the model equations, we calculate this using Matlab's “trapz” function, which employs the trapezoidal rule to compute the required quadrature.

Matlab scripts used for simulation and analyzing the results will be made available if requested.

## Supporting Information

Table S1 Glossary of variables for the basic model
(0.02 MB PDF)

Table S2 Constants
(0.03 MB PDF)

Table S3 Glossary of new variables for the intermediate and full models
(0.03 MB PDF)

Table S4 Additional constants for the intermediate and full model
(0.04 MB PDF)

Figure S1 Sensitivity analysis of the basic model. The basic model was simulated for 1440 min with five different frequencies of the exponential pulse profile of MAPKK: 8 min, 30 min, 60 min, 120 min and 240 min. Thereafter, each kinetic constant was varied by 10%, in turn, to visualize the effects of such fluctations to the overall frequency decoding ability of the system. Fold-differences of the accumulated concentrations for each subunit gene were then plotted. Only results for the kinetic constant, kcat1, have been shown here.
(0.16 MB PDF)

Figure S2 Sensitivity analysis of the expanded model without receptor dynamics. The expanded model without receptor dynamics, but with the inclusion of ERK5, was simulated for 1440 min with five different frequencies of the exponential pulse profile of MAPKK: 8 min, 30 min, 60 min, 120 min and 240 min. Thereafter, each kinetic constant related to ERK5 was varied by 10%, in turn, to visualize the effects of such fluctations to the overall frequency-decoding ability of the system. Fold-differences of the accumulated concentrations for each subunit-gene were then plotted. Only results for the kinetic constant, kcat1, have been shown here.
(0.16 MB PDF)

Figure S3 Sensitivity analysis of the expanded model with receptor dynamics to k1. The expanded model with receptor dynamics was simulated for 1440 min. Thereafter k1 was varied by 10% to visualize the effects of such fluctations to the overall frequency-decoding ability of the system. Fold-differences of the accumulated concentrations for each subunit gene were then plotted.
(0.16 MB PDF)

Figure S4 Sensitivity analysis of the expanded model with receptor dynamics to k11. The expanded model with receptor dynamics was simulated for 1440 min. Thereafter k11 was varied by 10% to visualize the effects of such fluctations to the overall frequency decoding ability of the system. Fold-differences of the accumulated concentrations for each subunit-gene were then plotted.
(0.16 MB PDF)

Figure S5 Sensitivity analysis of the expanded model with receptor dynamics to kinetic constants other than k1 and k11. The expanded model with receptor dynamics was simulated for 1440 min. Thereafter, each kinetic constant other than k1, k11 and those already tested, was varied by 10%, in turn, to visualize the effects of such fluctations to the overall frequency-decoding ability of the system. Fold-differences of the accumulated concentrations for each subunit-gene were then plotted. Only results for the kinetic constant, k3, have been shown here.
(0.16 MB PDF)

File S1 Supplementary information for methods and results
(0.10 MB PDF)
