# Supplementary material for: Negative Feedback Governs Gonadotrope Frequency-Decoding of Gonadotropin Releasing Hormone Pulse-Frequency
Source: PLoS One. 2009 Sep 29;4(9):e7244. doi: 10.1371/journal.pone.0007244 (PMC2746289; doi:10.1371/journal.pone.0007244)
Supplement: Table S1 — Glossary of variables for the basic model (0.02 MB PDF) [file pone.0007244.s001.pdf]

Variable	Description	Initial Concentration (nM)
MKK	MAPKK	0
pERK	Activated ERK1/2	0
pJNK	Activated JNK	0
pp38	Activated p38	0
DUSP1	MAPK phosphatase 1	0
DUSP4	MAPK phosphatase 2	0
α	αGSU mRNA	0
LHβ	LHβ mRNA	0
FSHβ	FSHβ mRNA	0
