# Supplementary material for: Negative Feedback Governs Gonadotrope Frequency-Decoding of Gonadotropin Releasing Hormone Pulse-Frequency
Source: PLoS One. 2009 Sep 29;4(9):e7244. doi: 10.1371/journal.pone.0007244 (PMC2746289; doi:10.1371/journal.pone.0007244)
Supplement: Table S2 — Constants (0.03 MB PDF) [file pone.0007244.s002.pdf]

Constant	Description/Value
$kcat_1$	Basic MAPK phosphorylation turnover number/9 min^{-1}<br>(from DOQCS pathway number 6, and verified in [7])
km_1	Basic MAPK phosphorylation Michaelis constant/46.2963 nM<br>(from DOQCS pathway number 6, and verified in [7])
$kcat_{-1}$	Basic MAPK dephosphorylation turnover number/60 min^{-1}<br>(from DOQCS pathway number 32, and verified in [7])
km_{-1}	Basic MAPK dephosphorylation Michaelis constant/66.667 nM<br>(from DOQCS pathway number 32, and verified in [7])
k_{f1}	Rate of ERK1/2-induced DUSP production/0.005 min^{-1}<br>(estimated and adjusted from DOQCS pathway number 35,<br>and taking into account levels of ERK1/2)
δ_1	Rate of DUSP1 degradation/0.024 min^{-1}<br>(estimated based on [8])
k_{f2}	Rate of JNK-induced DUSP production/0.005 min^{-1}<br>(estimated based on [8])
δ_2	Rate of DUSP4 degradation/0.012 min^{-1}<br>(estimated based on [8])
s_1	αGSU mRNA synthesis rate/1 min^{-1}<br>(arbitrarily chosen)
s_2	LHβ mRNA synthesis rate/0.01 nM^{-1} min^{-1}<br>(arbitrarily chosen, but taking in account the product of two MAPKs)
s_3	FSHβ mRNA synthesis rate/0.001 nM^{-2} min^{-1}<br>(arbitrarily chosen, but taking in account the product of three MAPKs)
ERK	Total ERK1/2/360 nM<br>(from DOQCS pathway number 6, and verified in [7])
JNK	Total JNK/300 nM<br>(estimated from total amount of ERK1/2, and unpublished observations)
p38	Total p38/240 nM<br>(estimated from total amount of ERK1/2, and unpublished observations)
κ	Constant governing exponential decay of MKK/0.333<br>(arbitrarily chosen to give gradual exponential decay)
