# Supplementary material for: Negative Feedback Governs Gonadotrope Frequency-Decoding of Gonadotropin Releasing Hormone Pulse-Frequency
Source: PLoS One. 2009 Sep 29;4(9):e7244. doi: 10.1371/journal.pone.0007244 (PMC2746289; doi:10.1371/journal.pone.0007244)
Supplement: Table S3 — Glossary of new variables for the intermediate and full models (0.03 MB PDF) [file pone.0007244.s003.pdf]

Variable	Description	Initial Concentration (nM) (from [11, 12])
H	GnRH	0
R	Free GnRH-R	0.01
HR	Hormone-receptor complex	0
HRRH	Hormone-receptor dimer complex	0
GQ	$G_{q/11}$ protein	0.1
E	Effector protein	0
IP3	Inositol 1,4,5-trisphosphate	0
CAC	Cytosolic Ca^{2+}	0
CAER	Endoplasmic reticular (ER) Ca^{2+}	20 μM
CHO	Fraction of open ER Ca^{2+} channels	0
pBMK	Activated pERK5	0
BMKSP	ERK5-specific phosphatase	0
