# Supplementary material for: Negative Feedback Governs Gonadotrope Frequency-Decoding of Gonadotropin Releasing Hormone Pulse-Frequency
Source: PLoS One. 2009 Sep 29;4(9):e7244. doi: 10.1371/journal.pone.0007244 (PMC2746289; doi:10.1371/journal.pone.0007244)
Supplement: Table S4 — Additional constants for the intermediate and full model (0.04 MB PDF) [file pone.0007244.s004.pdf]

Constant	Description/Value (from [11, 12], unless otherwise stated)
k_{f3}	Rate of ERK5-induced BMKSP production/0.005 min^{-1}
	(estimated based on kinetics of ERK1/2 activation)
δ_3	Rate of BMKSP degradation/0.012 min^{-1}
	(estimated based on kinetics of ERK1/2 activation)
$\hat{s}_3$	Revised FSHβ mRNA synthesis rate/0.0001 nM^{-3} min^{-1}
	(arbitrarily chosen, but taking in account the product of four MAPKs)
k_1	Rate of GnRH binding/2.5 nM^{-1} min^{-1}
k_{-1}	Rate of GnRH-release/5 min^{-1}
k_2	Rate of receptor dimerization/2500 nM^{-1} min^{-1}
k_{-2}	Rate of receptor monomerization/5 min^{-1}
k_3	Rate of effector protein production/4000 nM^{-1} min^{-1}
k_{-3}	Rate of release of GQ from HRRH/200 min^{-1}
k_5	Rate of IP3 production/2 x 10^7 min^{-1}
k_{-5}	Rate of IP3 degradation/10 min^{-1}
k_6	Constant governing rate of calcium release from ER/1 min^{-1}
k_{66}	Constant governing rate of calcium release from ER/10 μM^{-1} min^{-1}
k_{666}	Constant governing rate of calcium release from ER/0 μM^{-2} min^{-1}
k_{-6}	Rate of calcium pumped back to ER/5 min^{-1}
k_7	Rate of calcium pumped out of the cell/2.2 μM min^{-1}
k_8	Constant governing VGCC/0.4 nM^{-1} min^{-1}
k_{88}	Constant governing VGCC/0 μM^{-1} min^{-1}
k_{888}	Constant governing VGCC/0 μM^{-2} min^{-1}
k_9	Rate of calcium leaking into the cell/0.0002 min^{-1}
k_{11}	Basal rate of GnRH-R synthesis/8.3 x 10^{-5} nM min^{-1}
k_{-11}	Rate of GnRH-R degradation/8.3 x 10^{-4} min^{-1}
k_{33}	Rate of GQ increase by GnRH/2.7 min^{-1}
ERUL	Resting Ca^{2+} concentration in ER/40 μM
CAE	External Ca^{2+} concentration/1000 μM
α	Constant for fraction of open ER channels/2 nM^{-1}
β	Constant for fraction of open ER channel/4 min^{-1}
BMK	Total ERK5/50 nM
	(estimated based on total amount of ERK1/2 given by DOQCS, pathway number 6)
