# Supplementary figures and images for: Negative Feedback Governs Gonadotrope Frequency-Decoding of Gonadotropin Releasing Hormone Pulse-Frequency
Source: PLoS One. 2009 Sep 29;4(9):e7244. doi: 10.1371/journal.pone.0007244 (PMC2746289; doi:10.1371/journal.pone.0007244)

# Unperturbed Basic Model (Exponential Pulse)

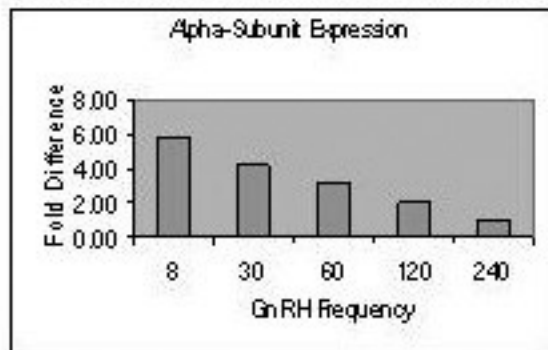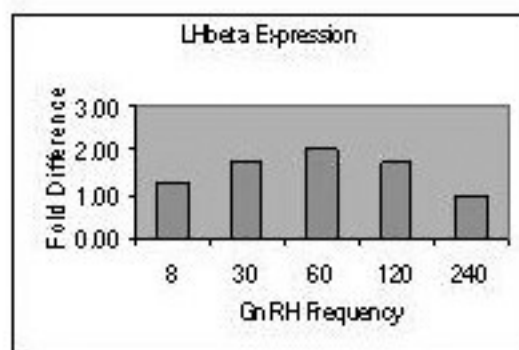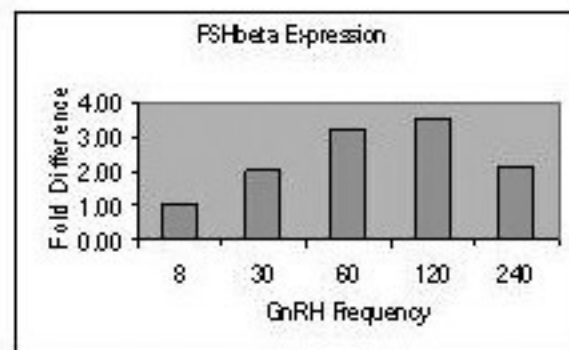

## Kcat1+10%

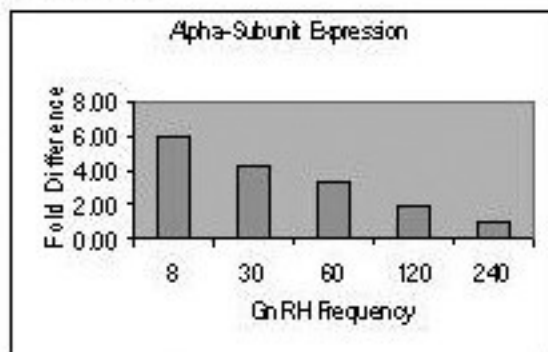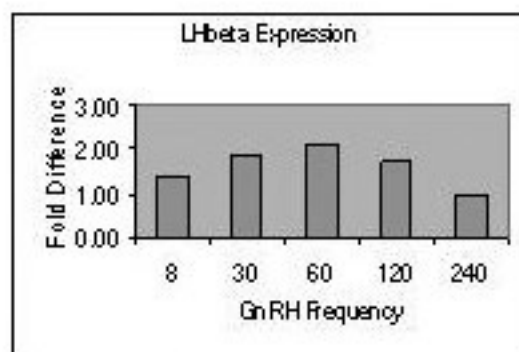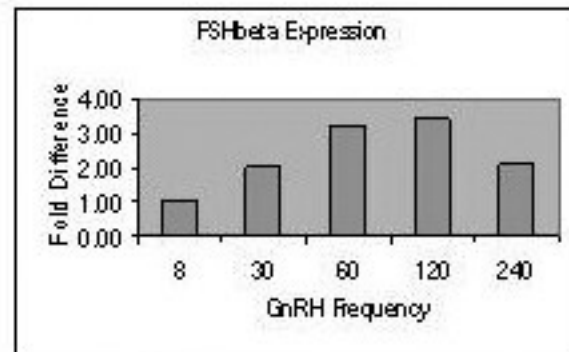

## Kcat1-10%

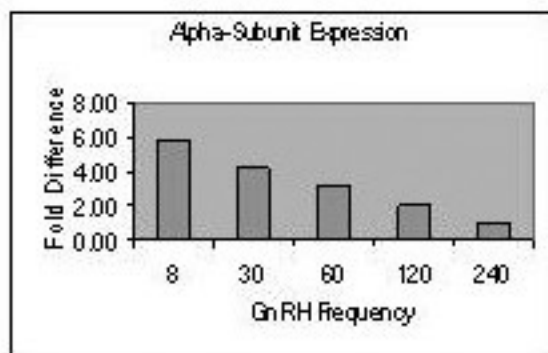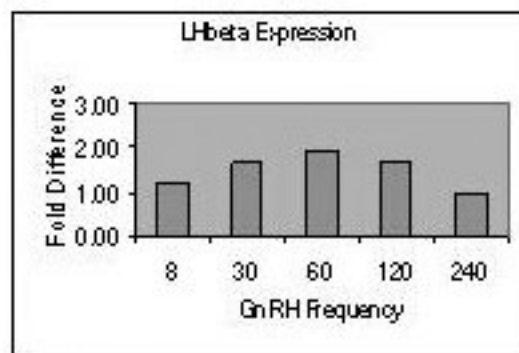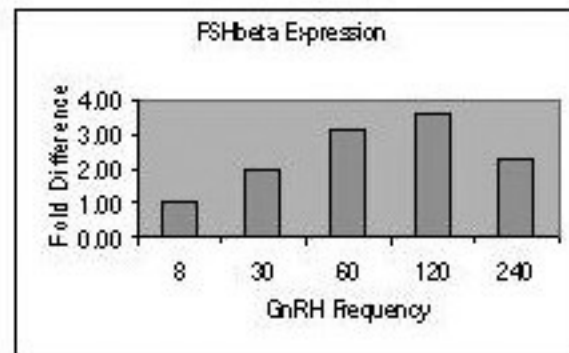

Supplement: Figure S1 — Sensitivity analysis of the basic model. The basic model was simulated for 1440 min with five different frequencies of the exponential pulse profile of MAPKK: 8 min, 30 min, 60 min, 120 min and 240 min. Thereafter, each kinetic constant was varied by 10%, in turn, to visualize the effects of such fluctations to the overall frequency decoding ability of the system. Fold-differences of the accumulated concentrations for each subunit gene were then plotted. Only results for the kinetic constant, kcat1, have been shown here. (0.16 MB PDF) [file pone.0007244.s005.pdf]

# Unperturbed Intermediate Model (Exponential Pulse)

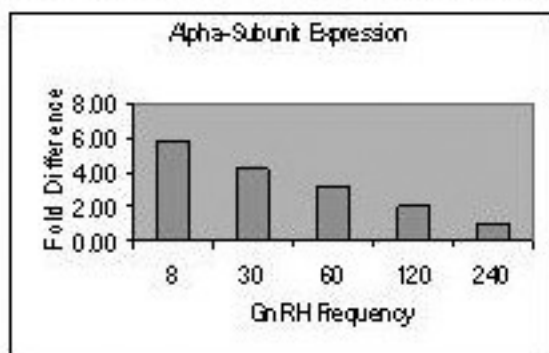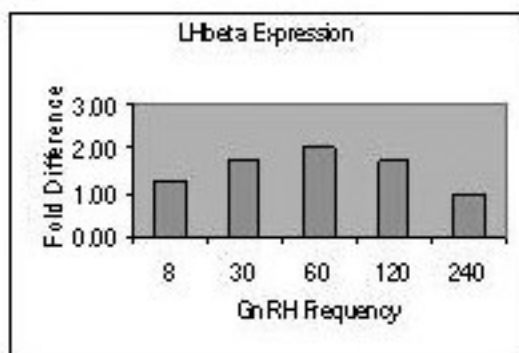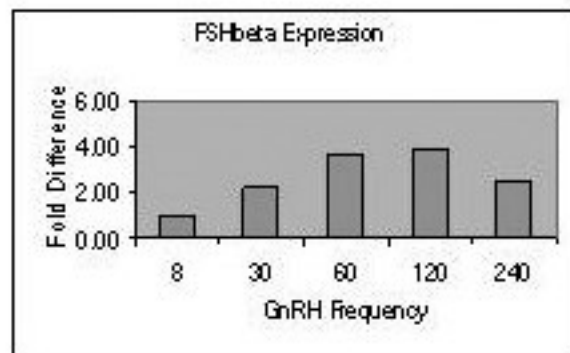

## Kcat1+10%

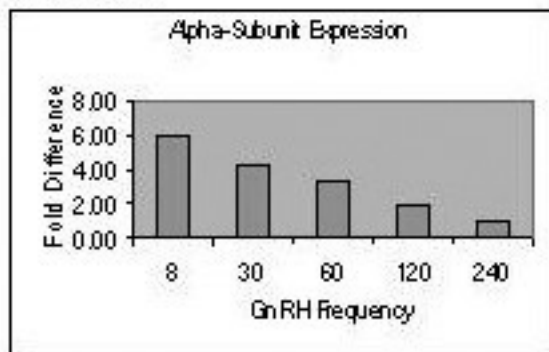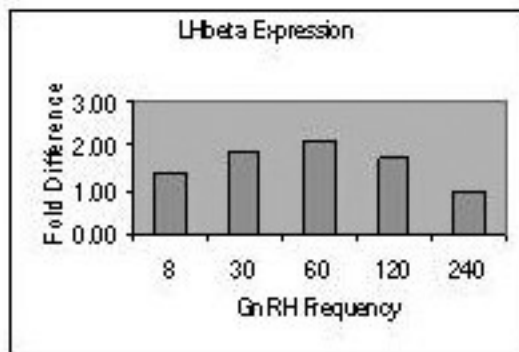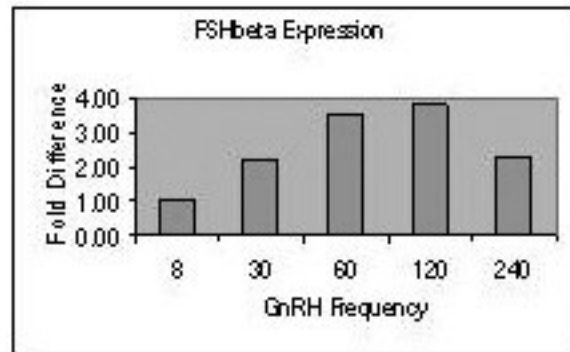

## Kcat1-10%

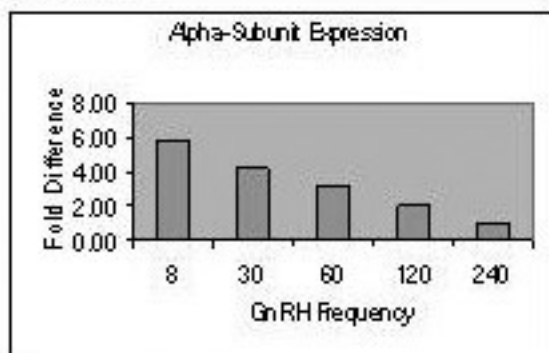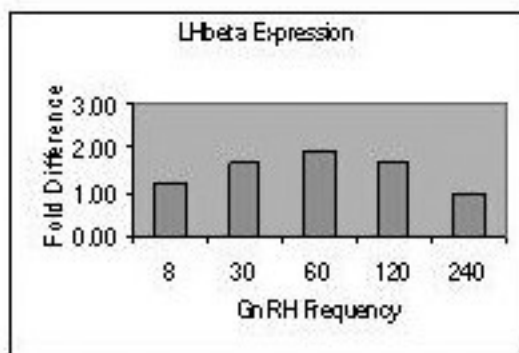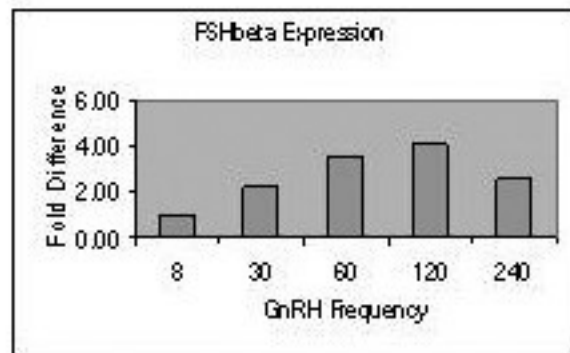

Supplement: Figure S2 — Sensitivity analysis of the expanded model without receptor dynamics. The expanded model without receptor dynamics, but with the inclusion of ERK5, was simulated for 1440 min with five different frequencies of the exponential pulse profile of MAPKK: 8 min, 30 min, 60 min, 120 min and 240 min. Thereafter, each kinetic constant related to ERK5 was varied by 10%, in turn, to visualize the effects of such fluctations to the overall frequency-decoding ability of the system. Fold-differences of the accumulated concentrations for each subunit-gene were then plotted. Only results for the kinetic constant, kcat1, have been shown here. (0.16 MB PDF) [file pone.0007244.s006.pdf]

# Unperturbed Full Model (Exponential Pulse)

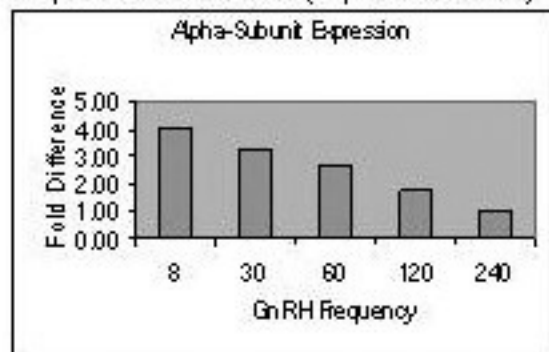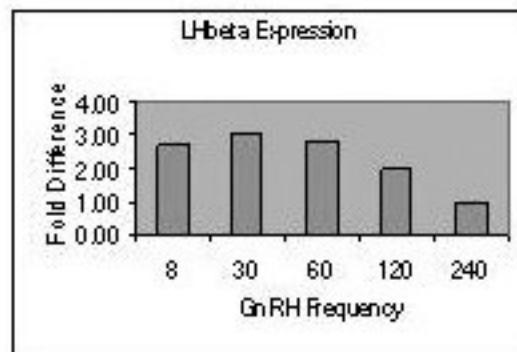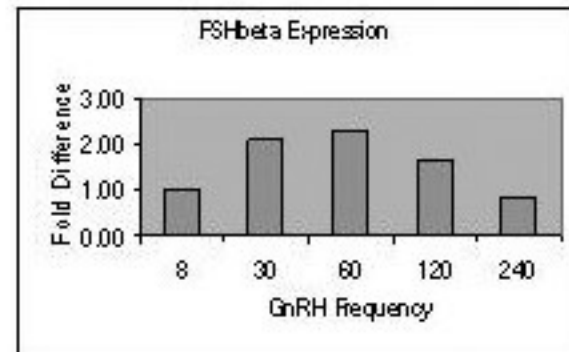

## K1+10%

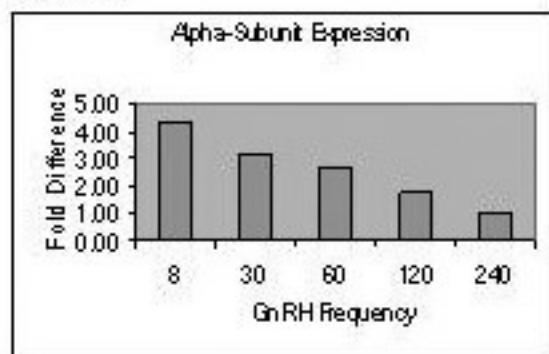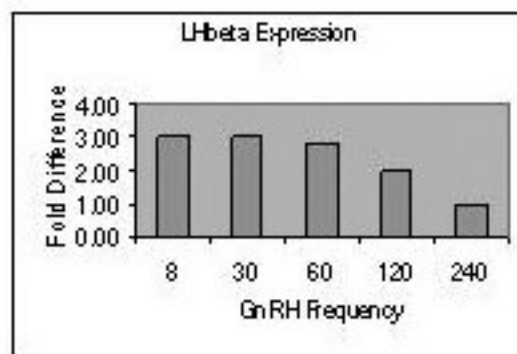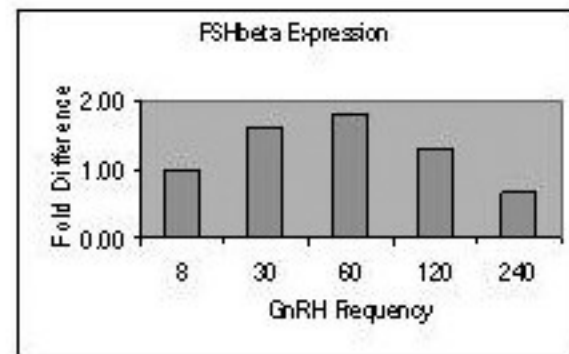

## K1-10%

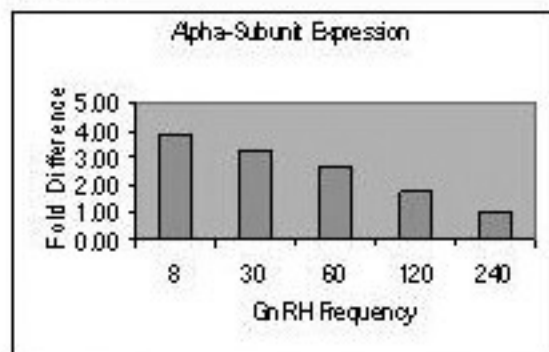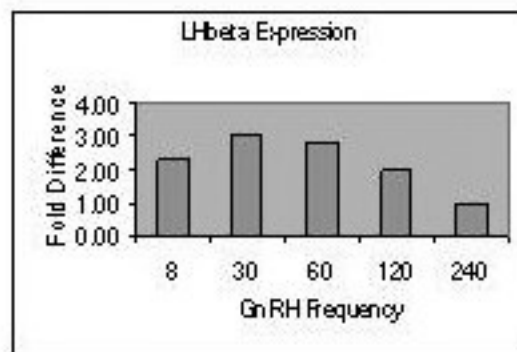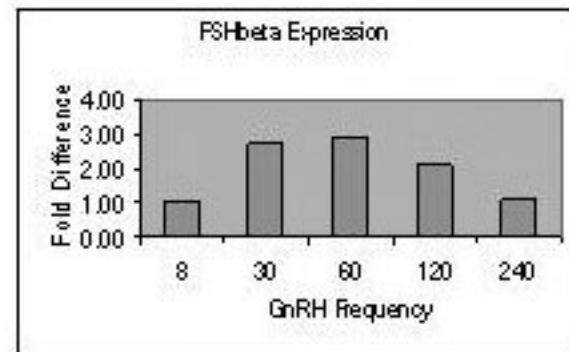

Supplement: Figure S3 — Sensitivity analysis of the expanded model with receptor dynamics to k1. The expanded model with receptor dynamics was simulated for 1440 min. Thereafter k1 was varied by 10% to visualize the effects of such fluctations to the overall frequency-decoding ability of the system. Fold-differences of the accumulated concentrations for each subunit gene were then plotted. (0.16 MB PDF) [file pone.0007244.s007.pdf]

# Unperturbed Full Model (Exponential Pulse)

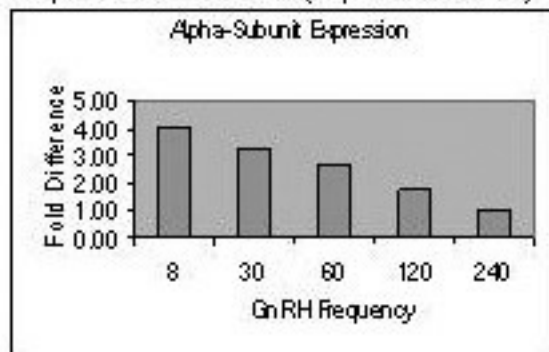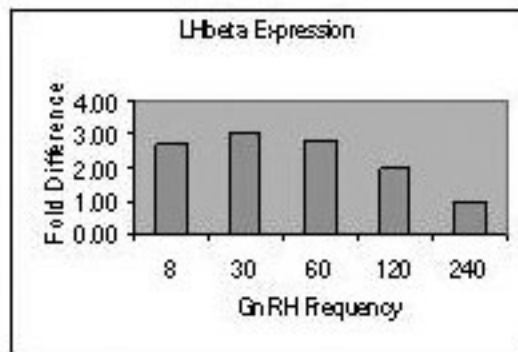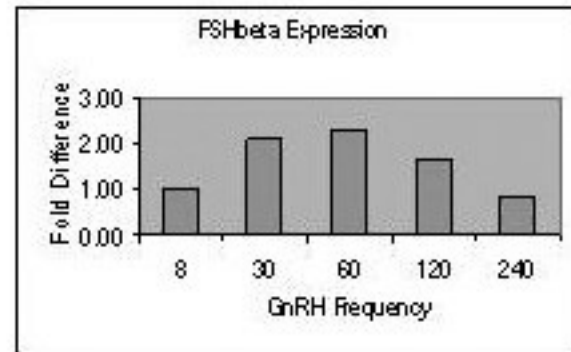

## K11+10%

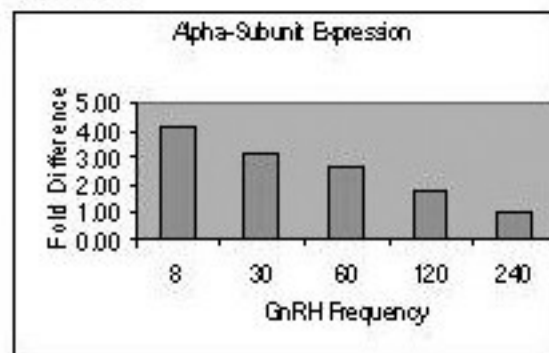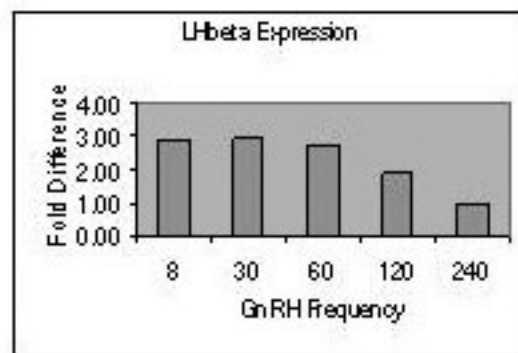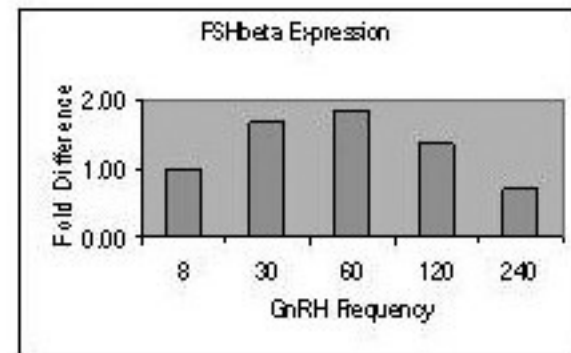

## K11-10%

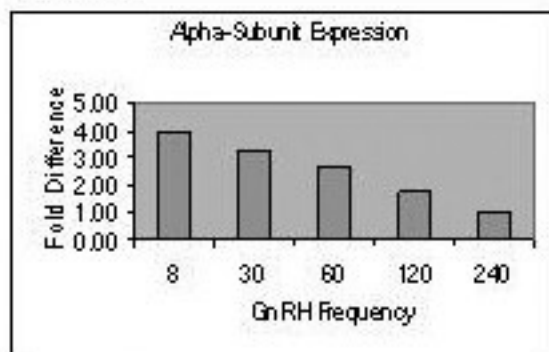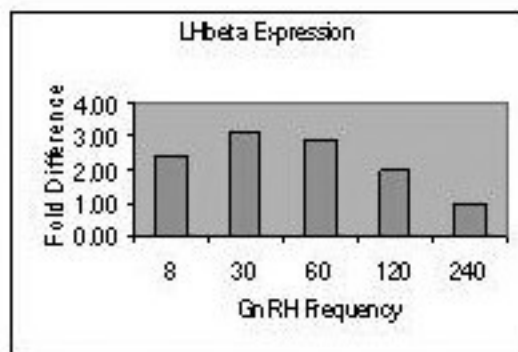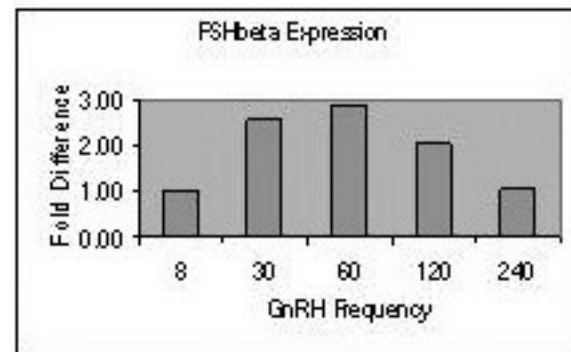

Supplement: Figure S4 — Sensitivity analysis of the expanded model with receptor dynamics to k11. The expanded model with receptor dynamics was simulated for 1440 min. Thereafter k11 was varied by 10% to visualize the effects of such fluctations to the overall frequency decoding ability of the system. Fold-differences of the accumulated concentrations for each subunit-gene were then plotted. (0.16 MB PDF) [file pone.0007244.s008.pdf]

# Unperturbed Full Model (Exponential Pulse)

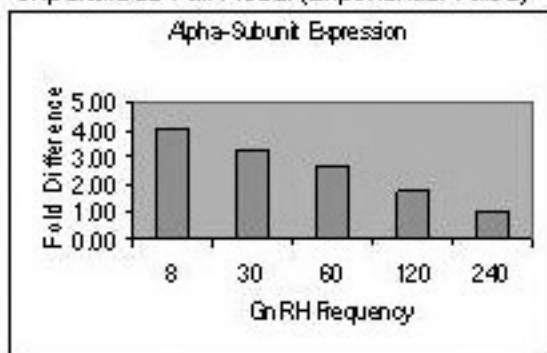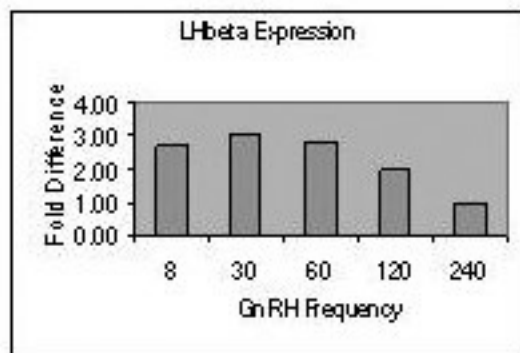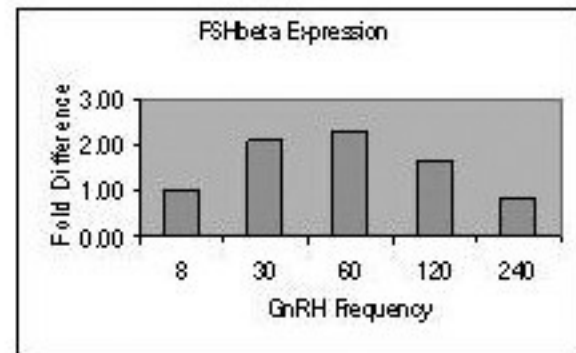

## K3+10%

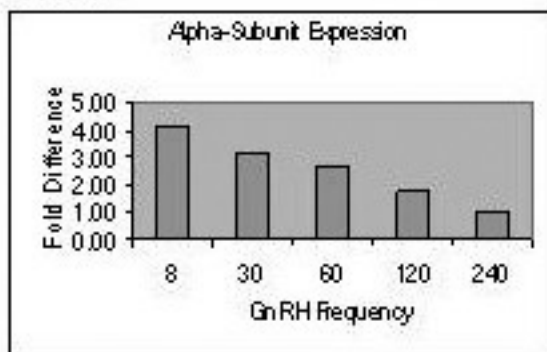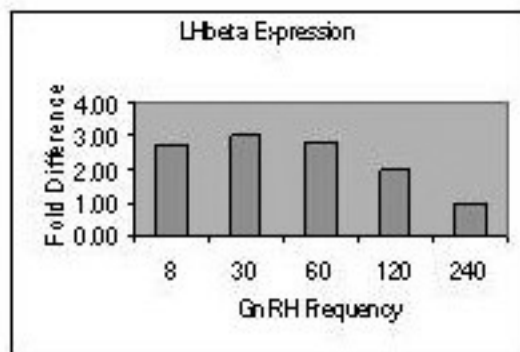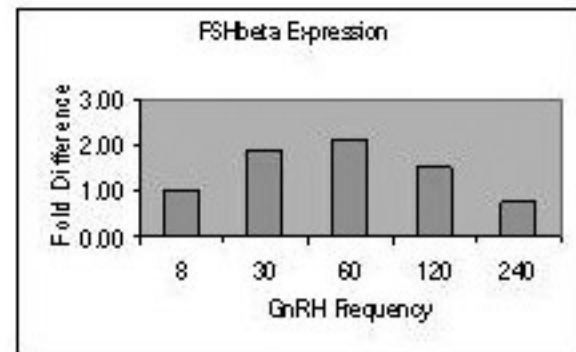

## K3-10%

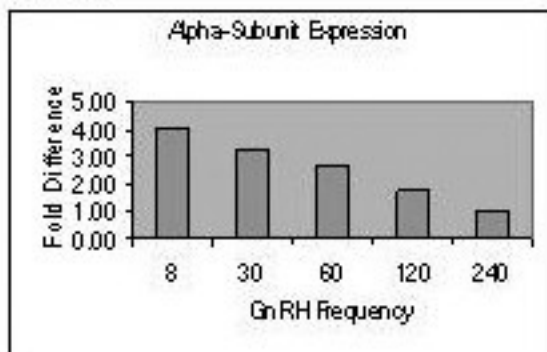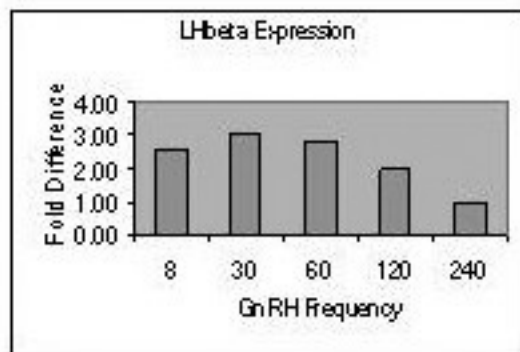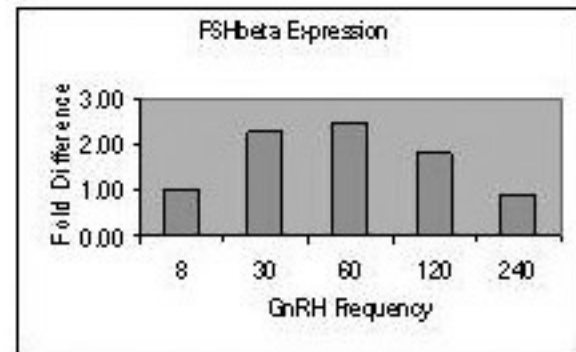

Supplement: Figure S5 — Sensitivity analysis of the expanded model with receptor dynamics to kinetic constants other than k1 and k11. The expanded model with receptor dynamics was simulated for 1440 min. Thereafter, each kinetic constant other than k1, k11 and those already tested, was varied by 10%, in turn, to visualize the effects of such fluctations to the overall frequency-decoding ability of the system. Fold-differences of the accumulated concentrations for each subunit-gene were then plotted. Only results for the kinetic constant, k3, have been shown here. (0.16 MB PDF) [file pone.0007244.s009.pdf]
