# Supplementary material for: Negative Feedback Governs Gonadotrope Frequency-Decoding of Gonadotropin Releasing Hormone Pulse-Frequency
Source: PLoS One. 2009 Sep 29;4(9):e7244. doi: 10.1371/journal.pone.0007244 (PMC2746289; doi:10.1371/journal.pone.0007244)
Supplement: File S1 — Supplementary information for methods and results (0.10 MB PDF) [file pone.0007244.s010.pdf]

Negative feedback governs gonadotrope frequency-decoding of gonadotropin releasing hormone pulse-frequency (Supplementary information)

Stefan Lim, Lilach Pnueli, Jinghui Tan, Zvi Naor,
Gunaretnam Rajagopal, and Philippa Melamed

Supplementary materials and Methods

The basic model

GnRH, upon binding its receptor, triggers a series of signal transduction processes, resulting in the activation of the three major MAPKs: ERK1/2, JNK and p38 [1–4]. Each of these MAPKs figures prominently in the expression of one, two or all three of the gonadotropin-subunit genes. Using this simple idea, we can formulate a series of ordinary differential equations to describe this process, involving the molecular species given in Supplementary Table 1.

The detailed signal transduction events that occur after receptor-binding by the ligand up to the activation of each MAPK only determines the kinetics and strength of the activation of each MAPKs, and therefore need not be modeled in detail. Since modeling this process will unnecessarily increase the level of complexity and simulation time, one would choose rather to treat the activation of each of the three MAPKs as dependent directly on the GnRH stimulus through first-order Michaelis-Menten kinetics. For this, one assumes that each activating kinase, MAPKK, has an activation profile mimicking that of the pulsatile GnRH stimulus, differing only in amplitude. We also assume that the phosphatases involved act directly at the level of the MAPK and not the MAPKK [5, 6]. This would be sufficient to mimic the qualitative behavior of the system.

Each activating MAPKK acts on an unphosphorylated MAPK to yield the

phosphorylated pMAPK. Applying first-order Michaelis-Menten kinetics with turnover number $kcat_1$, and Michaelis constant, km_1, one can represent this by:

$$\frac{d[pMAPK]}{dt} = \frac{kcat_1[MAPKK]([MAPK]-[pMAPK])}{km_1+([MAPK]-[pMAPK])},$$

where ([MAPK] - [pMAPK]) denotes the amount of unphosphorylated MAPK remaining at any one time. Similarly, the phosphorylated pMAPK is dephosphorylated by the relevant phosphatase MKP. Again, using first-order Michaelis-Menten kinetics with turnover number $kcat_{-1}$, and Michaelis constant, km_{-1}, one represents this by:

$$\frac{d[pMAPK]}{dt} = -\frac{kcat_{-1}[MKP][pMAPK]}{km_{-1}+[pMAPK]}.$$

Combining the phosphorylation and dephosphorylation steps, we thus have, for each of the three MAPKs, the following equations:

$$\frac{d[pERK]}{dt} = \frac{kcat_1[MKK(t)]([ERK]-[pERK])}{km_1+([ERK]-[pERK])} - \frac{kcat_{-1}[DUSP1][pERK]}{km_{-1}+[pERK]};$$

$$\frac{d[pJNK]}{dt} = \frac{\frac{[JNK]}{[ERK]}kcat_1[MKK(t)]([JNK]-[pJNK])}{\frac{[JNK]}{[ERK]}km_1+([JNK]-[pJNK])} - \frac{kcat_{-1}[DUSP4][pJNK]}{km_{-1}+[pJNK]};$$

$$\frac{d[pp38]}{dt} = \frac{\frac{[p38]}{[ERK]}kcat_1[MKK(t)]([p38]-[pp38])}{\frac{[p38]}{[ERK]}km_1+([p38]-[pp38])} - \frac{kcat_{-1}[DUSP1][pp38]}{km_{-1}+[pp38]},$$

where each constant is explained in Supplementary Table 2.

The values of the $kcat_1$, km_1, $kcat_{-1}$ and km_{-1} have all been adapted from the *Database of Quantitative Cellular Signaling* (DOQCS) [7], as the basic kinetic constants for the phosphorylation and de-phosphorylation of ERK. Since the data given by DOQCS hold good for brain cells and for neural signaling [7], it is envisaged that the kinetics of similar reactions in the gonadotropes would not vary

significantly. Hence, such adaptations are acceptable. To account for the differences in the relative amounts of the activating kinases, as well as the possible differences in the Michaelis constants, we have scaled both the amount of each activating kinase, as well as its Michaelis constant, by a factor $\frac{[MAPK_{total}]}{[ERK_{total}]}$. This is because in the gonadotropes, of the three MAPKs, ERK1/2 is present in the highest amounts (Seger R and Lawson MA, pers. commun.) and the kinetic constants were chosen with ERK1/2 as the basis. We do not have to carry out this scaling for the dephosphorylation terms, because each phosphatase has been uniquely represented and described. The typical initial cellular concentration of ERK1/2 (360 nM) has also been adapted from DOQCS. Initial concentrations of JNK and p38 have been estimated based on unpublished observations (Lawson MA, unpublished). The maximum concentration of the active MAPKK for ERK1/2 has been arbitrarily set at 50 nM, which is about four times less than that given for a generic MAPK (180 nM) in DOQCS. The rationale behind this choice was that in a MAPK cascade, the upstream kinases ought to be present in increasingly smaller amounts for the cascade to exhibit ultra-sensitivity, as a result of which the steepness of the shape of the MAPK stimulus/response curve makes the cascade particularly appropriate for mediating different cellular processes, allowing a cell to switch from one discrete state to another almost instantaneously [8].

The phosphatases are up-regulated by their respective kinases as documented in the literature, and this is expressed as simple proportions of these kinases. The basic rate of DUSP1 activation has been taken from DOQCS. Moreover, as the induction of DUSP4 is much slower as compared to DUSP1 [9], the rate of DUSP4 induction by ERK1/2 is reduced to 20% that of DUSP1. Their degradation is proportional to their instantaneous amounts. This gives:

$$\frac{d[DUSP1]}{dt} = k_{f1}[pERK] + k_{f2}[pJNK] - \delta_1[DUSP1];$$

$$\frac{d[DUSP4]}{dt} = (0.2)k_{f1}[pERK] + k_{f2}[pJNK] - \delta_2[DUSP4].$$

The rate of change of the amounts of each gonadotropin-subunit mRNA is made proportional to the product of the amounts of their requisite MAPKs. This will allow one to test whether GnRH frequencies indeed synchronize the periods of highest activity for the various MAPKs for optimal subunit expression. If this were not the case, and these MAPKs were asynchronously-activated, then the

product of their amounts would remain relatively stable with time, without peaking significantly. The consequence of this would be the lack of unique frequency regimes where each gonadotropin-subunit is optimally expressed. We thus have:

$$\begin{aligned}
\frac{d[\alpha]}{dt} &= s_1[pERK]; \\
\frac{d[LH\beta]}{dt} &= s_2[pERK][pJNK]; \\
\frac{d[FSH\beta]}{dt} &= s_3[pERK][pJNK][pp38],
\end{aligned}$$

where s_1, s_2 and s_3 have all be arbitrarily chosen, without any ill-effect on the overall behavior of each gonadotropin-subunit gene.

Degradation of these products was disregarded so that one would be able to observe their accumulation, as a measure of the transcriptional power of the MAPKs. Degradation terms could potentially mask the full-extent of this power, and so have been omitted. This is also consistent with the promoter assays carried out previously [10], which reveal only the transcriptional strength of each promoter under the induction of pulsatile GnRH, and not the mechanics of gonadotropin-subunit mRNA regulation.

The expanded models

To expand the basic model to include the ERK5 induction of FSHβ and its negative-regulation of the GnRH-R, we introduce a number of new variables representing these components (Supplementary Table 3), together with the additional rate constants (Supplementary Table 4).

The basic model is expanded in two stages. In the first, we add the ERK5 induction of FSHβ. Since there is no published data on a ERK5-specific phosphatase, we will have to assume the existence of one. This is reasonable, based on how specific-phosphatases already exist for the common MAPKs [5,6]. Also, since we do not as yet understand how this ERK5-specific phosphatase would be regulated, we assume that it is positively-regulated by ERK5, as is the case again with other known-MAPKs. Therefore, one has, as before:

$$\frac{d[pBMK]}{dt} = \frac{\frac{[BMK]}{[ERK]}kcat_1[MKK(t)]([BMK]-[pBMK])}{\frac{[BMK]}{[ERK]}km_1+([BMK]-[pJNK])} - \frac{kcat_{-1}[BMKSP][pBMK]}{km_{-1}+[pBMK]};$$

$$\frac{d[BMKSP]}{dt} = k_{f3}[pBMK] - \delta_3[BMKSP].$$

To complete this model, we then modify the expression for FSHβ to include ERK5:

$$\frac{d[FSH\beta]}{dt} = \hat{s}_3[pERK][pJNK][pp38][pBMK],$$

where s_3 has been re-scaled to $\hat{s}_3$ to fit in the fourth variable. Again, the concentration of ERK5 has been estimated similar to JNK and p38, while the kinetic constants of the BMKSP, the ERK5-specific phosphatase, have been arbitrarily made identical to those of DUSP4. This model thus allows us to test the ERK5-effect on FSHβ in the frequency decoding process.

Finally, to complete the expansion of the basic model to include receptor dynamics, we append the signaling components upstream of the MAPKKs, beginning from the GnRH-R. Based on the ideas of Conn *et al.* [11,12] together with their kinetic parameters, we similarly assume that GnRH binds to the receptors

via a simple reversible reaction:

$$\mathrm{H + R} \underset{k-1}{\overset{k1}{\rightleftharpoons}} \mathrm{HR}.$$

We furnish the replenishment of degraded receptors with a basal rate of $k_{11} = 8.3$ x 10^{-6} nM min^{-1}, and degradation of free receptors, R, with a rate of $k_{-11} = 8.3$ x 10^{-4} min^{-1}, where degradation but not synthesis of R is proportional to the amount of free receptors, R [12]. Since JNK up-regulates the levels of GnRH-R [13, 14], we account for the induction of R synthesis by multiplying k_{11} by a factor $(1 + \varepsilon \cdot \frac{[pJNK]}{[JNK]})$, so that the more JNK is activated, the greater the induction of R.

On the other hand, Nur77 has been shown to down-regulate GnRH-R expression [15]. Since ERK5 activates Nur77 in T-cells, and there is thus a possibility of ERK5 down-regulating GnRH-R expression levels, we describe this impediment of GnRH-R synthesis by multiplying k_{11} by a second factor $(1 - \gamma \cdot \frac{[pBMK]}{[BMK]})$. Clearly, the more highly ERK5 is activated, the closer $\frac{[pBMK]}{[BMK]}$ is to unity, so that the overall rate of synthesis is significantly reduced.

Both ε and γ are parameters that take the value 0 or 1, to allow us to examine the roles of JNK and ERK5 more easily in influencing gonadotropin-subunit gene expression by directly affecting GnRH-R levels. Furthermore, as very likely $(1 - \gamma \cdot \frac{[pBMK]}{[BMK]}) << 1$ for $\gamma = 1$, we increase k_{11} by one magnitude to 8.3 x 10^{-5}, so that the levels of GnRH-R will be amply replenished.

The ligand-bound receptor *HR* then homodimerizes according to:

$$\mathrm{HR + HR} \underset{k-2}{\overset{k2}{\rightleftharpoons}} \mathrm{HRRH}.$$

The G-protein, GQ, reacts with the dimerized receptor, HRRH, to produce an effector, E. In this context, E represents PLC.

$$\mathrm{HRRH + GQ} \underset{k-3}{\overset{k3}{\rightleftharpoons}} \mathrm{E}.$$

The number of membrane associated GQ proteins increases in response to a GnRH agonist [12, 16], and for simplicity, we assume that the increase of GQ proteins near the membrane depends on the concentration of HRRH in the membrane. The kinetic coefficient k_{33} is the parameter determining the rate of increase of GQ at the membrane in response to the formation of HRRH. This increase is only

significant for the first 20 minutes, and thereafter, any increase is negligible [16]. Hence, we multiply this k_{33} by $e^{-t/20}$ to model this loss of significance [12].

From these biochemical equations, we derive, using mass-action kinetics, the following first-order differential equations:

$$\begin{aligned}
\frac{d[R]}{dt} &= -k_1[H][R]+k_{-1}[HR]+k_{11}[pJNK]-k_{-11}[pBMK];\\
\frac{d[HR]}{dt} &= k_1[H][R]-k_{-1}[HR]+2k_{-2}[HRRH]-2k_2[HR][HR];\\
\frac{d[HRRH]}{dt} &= -k_{-2}[HRRH]+k_2[HR][HR]-k_3[GQ][HRRH]+k_{-3}[E];\\
\frac{d[GQ]}{dt} &= -k_3[GQ][HRRH]+k_{-3}[E];\\
\frac{d[E]}{dt} &= k_3[GQ][HRRH]-k_{-3}[E].
\end{aligned}$$

The production of IP_3 is then assumed to be proportional to the concentration of E [11, 12]. Moreover, we assume that IP_3 is converted to inactive metabolites at a rate proportional to its concentration, thus avoiding the complexities of IP_3 metabolism:

$$\frac{d[IP3]}{dt} = k_5[E]-k_{-5}[IP3].$$

To model calcium dynamics, we first note that Ca^{2+} is stored in the ER and is released when IP_3 binds to the receptors on the ER. Without modeling the specific dynamics of the IP_3-binding, we assume that the fraction of open channels (CHO) allowing calcium to be released into the cytosol is defined by:

$$CHO(t) = \left[\frac{\alpha 10^{-3}[IP_3(t)]}{1+\alpha 10^{-3}[IP_3(t)]}\right](0.3+0.3\beta t_p e^{1-\beta t_p}),$$

which depends on IP_3 concentration in a Michaelis-Menten-type saturating fashion [11]. To allow for pulsatility of the GnRH stimulus, t_p refers to the time after the start of each pulse. When α = 2 nM^{-1} and β = 4 min^{-1}, then the fraction of open channels reaches its maximum 0.25 min after the start of each pulse, consistent with published data [17].

We then assume that the release of Ca^{2+} from the ER is governed by the rate constant ERR given by:

$$ERR = k_6 + k_{66}[CAC] - k_{666}[CAC]^2.$$

This reflects the fact that the release is induced by low cytosolic Ca^{2+}-levels (through the parameter k_{66}), and inhibited by high cytosolic Ca^{2+}-levels (via parameter k_{666}). Hence, the rate of Ca^{2+}-release from the ER is jointly proportional to the fraction of open channels and the calcium gradient between the ER and the cytosol, with rate constant ERR:

$$[calcium\ release] = ERR \cdot CHO \cdot ([CAER] - [CAC]).$$

At the same time, Ca^{2+} is pumped back into the ER at a rate jointly proportional to CAC (via a Michaelis-Menten type expression with Hill's coefficient = 2) and the difference between the resting concentration of Ca^{2+} in the ER, ERUL, and CAER [11]. Thus,

$$\begin{aligned}\frac{d[CAER]}{dt} &= -ERR \cdot CHO \cdot ([CAER] - [CAC]) \\ &\quad + k_{-6}\frac{2[CAC]^2}{0.5 + 2[CAC]^2}([ERUL] - [CAER]).\end{aligned}$$

The dynamics of CAC, based purely on ER-regulation is given by:

$$\begin{aligned}\frac{d[CAC]}{dt} &= (0.05)ERR \cdot CHO \cdot ([CAER] - [CAC]) \\ &\quad -(0.05)k_{-6}\frac{2[CAC]^2}{0.5 + 2[CAC]^2}([ERUL] - [CAER]),\end{aligned}$$

where the factor 0.05 takes into account the calcium flux from the cytosol to the ER, assumed to be $\frac{1}{20}$ the volume of the cell, or *vice versa*. The effector E also activates VGCCs in the cell membrane [18]. Denoting CAE to be the Ca^{2+} in the external medium of constant concentration, the calcium influx through VGCCs is assumed to be proportional to the gradient ([CAE]-[CAC]). From [11], the rate constant, VSR, for this influx takes the form:

$$VSR \quad = \quad k_8[E] + k_{88}[CAC] - k_{888}[CAC]^2,$$

because of evidence that this rate is facilitated by low concentrations of CAC, but inhibited by high concentrations [18]. Finally, calcium is pumped out of the cell using second-order Michaelis-Menten kinetics and Ca^{2+} leakage into the cell is considered a simple first-order process. Hence, the dynamics of CAC can be re-expressed as:

$$\begin{aligned}\frac{d[CAC]}{dt} \quad = \quad & (0.05)ERR \cdot CHO \cdot ([CAER] - [CAC]) \\ & -(0.05)k_{-6}\frac{2[CAC]^2}{0.5 + 2[CAC]^2}([ERUL] - [CAER]) \\ & + VSR \cdot ([CAE] - [CAC]) - \frac{k_7[CAC]^2}{0.1 + [CAC]^2} + k_9[CAE],\end{aligned}$$

to include the other regulatory processes.

To bridge this addendum to the basic model, we assume that MKK follows the same activation profile as CAC. This is reasonable, in view of the fact that CAC activates PKC, which is the upstream activator of the various MAPK cascades in gonadotrope cells [1]. Nevertheless, because [CAC] ranges between 0.1 and 1 μM, we multiply it by a factor of 50 and re-assign its unit as nM to convert [CAC] to [MKK] of the basic model. Alternatively, we can co-multiply [CAC] by 50 nM and 1 μM^{-1} to effect the same conversion, but without the need for a re-assignment of units.

Supplementary Results

Sensitivity analysis of the basic model

To determine if the basic model is robust and whether the positive results obtained were unique to a single set of parameter values, a sensitivity analysis was carried out using both GnRH profiles for the model with and without negative feedback. Each kinetic parameter was adjusted in turn by 10% of its original value, and the trends of gonadotropin-subunit expression with various frequencies were noted as before.

Distinct differential gene expression was maintained throughout the changes in each of the kinetic constants perturbed for the model with feedback (Supplementary Figure 1). Only the results of changes in $kcat_1$ are shown. However, the other kinetic constants also behave in a like manner. Similarly, the lack of differential gene expression was observed for all variations of each kinetic constant for the model without feedback (data not shown).

Therefore the basic model was deemed to be robust, and the positive results obtained were not unique to a single set of parameter values.

Sensitivity analysis of the expanded model without receptor dynamics

To ascertain the robustness of the expanded model without receptor dynamics, a sensitivity analysis was again carried out. Since this model is the basic model augmented with ERK5 dynamics, and as ERK5 behaves autonomously in this model, only a sensitivity analysis of all kinetic parameters pertaining to ERK5 and not the others, needed to be done. Again, adjusting each of these rate constants by 10% did not affect the overall ability of the system to demonstrate differential gene expression, confirming the robustness of the intermediate model. As with the basic model, only the results of changes in $kcat_1$ are shown, with the other kinetic constants behaving in a like manner (Supplementary Figure 2).

Sensitivity analysis of the expanded model with receptor dynamics

Finally, a sensitivity analysis was carried out purely on the parameters pertaining to the receptor module of the model expanded to include receptor dynamics,

with the rest already been shown to be robust. For k_1 and k_{11}, fluctuations within 10% of their given values caused LHβ expression to peak at both 8 and 30 min pulse frequencies (Supplementary Figures 3, 4). This deviated marginally from the singular peak frequency of 30 min for optimal LHβ expression in the unperturbed model. Nevertheless, in spite of the broadening of the optimal frequency spectrum for LHβ expression, changes in k_1 and k_{11} did not affect the overall ability of the system to demonstrate differential gene expression. On the other hand, the full model was found to be stable against fluctuations in the values of all the other rate constants. The three distinct frequencies of 8 min for α-subunit, 30 min for LHβ and 60 min for FSHβ peak-expression were maintained throughout the perturbations (only the results for k_3 are shown (Supplementary Figure 5)).

References

[1] Naor Z, Bernard O, and Seger R. Activation of MAPK cascades by G-protein-coupled receptors: The case of gonadotropin-releasing hormone receptor. *Trends Endocrinol. Metab.*, 11:91–99, 2000.

[2] Harris D, Bonfil D, Chuderland D, Kraus S, Seger R, and Naor Z. Activation of MAPK cascades by GnRH: ERK and Jun N-terminal kinase are involved in basal and GnRH-stimulated activity of the glycoprotein hormone LHβ-subunit promoter. *Endocrinology*, 143:1018–1025, 2002.

[3] Harris D, Chuderland D, Bonfil D, Kraus S, Seger R, and Naor Z. Extracellular signal-regulated kinase and c-src, but not Jun N-terminal kinase, are involved in basal and gonadotropin-releasing hormone-stimulated activity of the glycoprotein hormone α-subunit promoter. *Endocrinology*, 144:612–622, 2003.

[4] Bonfil D, Chuderland D, Kraus S, Shahbazian D, Friedberg I, Seger R, and Naor Z. Extracellular signal-regulated kinase, Jun N-terminal kinase, p38 and c-src are involved in gonadotropin-releasing hormone-stimulated activity of the glycoprotein hormone follicle-stimulating hormone β-subunit promoter. *Endocrinology*, 145:2228–2244, 2004.

[5] Jeffrey KL, Camps M, Rommel C, and Mackay CR. Targeting dual-specificity phosphatases: manipulating MAP kinase signaling and immune responses. *Nat. Rev. Drug Discov.*, 6:392–403, 2007.

[6] Owens DM and Keyse SM. Differential regulation of MAP kinase signalling by dual-specificity protein phosphatases. *Oncogene*, 26:3203–3213, 2007.

[7] Bhalla US and Iyengar R. Emergent properties of networks of biological signaling pathways. *Science*, 283:381–387, 1999.

[8] Huang CF and Ferrell JE. Ultrasensitivity in the mitogen-activated protein kinase cascade. *Proc. Natl. Acad. Sci. USA*, 93:10078–10083, 1996.

[9] Zhang T and Roberson MS. Role of MAP kinase phosphatases in GnRH-dependent activation of MAP kinases. *J. Mol. Endocrinol.*, 36:41–50, 2006.

[10] Bedecarrats GY and Kaiser UB. Differential regulation of gonadotropin subunit-gene promoter activity by pulsatile gonadotropin-releasing hormone (GnRH) in perifused LβT2 cells: role of GnRH receptor concentration. *Endocrinology*, 144:1802–1811, 2003.

[11] Blum JJ, Reed MC, Janovick JA, and Conn PM. A mathematical model quantifying GnRH-induced LH secretion from gonadotropes. *Am. J. Physiol. Endocrinol. Metab.*, 278:263–272, 2000.

[12] Washington TM, Blum JJ, Reed MC, and Conn PM. A mathematical model for LH release in response to continuous and pulsatile exposure of gonadotrophs to GnRH. *Theoret. Biol. and Med. Model.*, 1:9, 2004.

[13] Ellsworth BS, White BR, Burns AT, Cherrington BD, Otis AM, and Clay CM. c-Jun N-terminal kinase activation of activator protein-1 underlies homologous regulation of the gonadotropin-releasing hormone receptor gene in αT3-1 cells. *Endocrinology*, 144:839–849, 2003.

[14] White BR, Duval DL, Mulvaney JM, Roberson MS, and Clay CM. Homologous regulation of the gonadotropin-releasing hormone receptor gene is partially mediated by protein kinase C activation of an activator protein-1 element. *Mol. Endocrinol.*, 13:566–577, 1999.

[15] Sadie H, Styger G, and Hapgood J. Expression of the mouse gonadotropin-releasing hormone receptor gene in αT3-1 gonadotrope cells is stimulated by cyclic 3', 5'-adenosine monophosphate and protein kinase A, and is modulated by steroidogenic factor-1 and Nur 77. *Endocrinology*, 144:1958–1971, 2003.

[16] Cornea A, Jankovic JA, Stanislaus D, and Conn PM. Redistribution of $G_{q/11}\alpha$ in the pituitary gonadotrope in response to a gonadotropin-releasing hormone agonist. *Endocrinology*, 139:397–402, 1998.

[17] Ramos-Franco J, Fill M, and Mignery GA. Isoform-specific function of single inositol 1,4,5-triphosphate receptor channels. *Biophys. J*, 75:834–839, 1998.

[18] Stojilkovic SS, Iida T, Merelli F, and Catt KJ. Calcium signaling and secretory responses in endothelin-stimulated anterior pituitary cells. *Mol. Pharmacol.*, 39:762–770, 1991.
